# Supplementary material for: Taxon-specific aerosolization of bacteria and viruses in an experimental ocean-atmosphere mesocosm
Source: Nat Commun. 2018 May 22;9:2017. doi: 10.1038/s41467-018-04409-z (PMC5964107; doi:10.1038/s41467-018-04409-z)
Supplement: Supplementary file 1 — Supplementary Information [file 41467_2018_4409_MOESM1_ESM.pdf]

Supplementary Information

Taxon-specific aerosolization of bacteria and viruses in an experimental ocean-atmosphere mesocosm

Michaud *et al.*

1. **Supplementary Figures**
2. **Supplementary Tables**
3. **Supplementary Notes**

   Supplementary Note 1: Bloom dynamics

   Supplementary Note 2: Genomic data analysis

      DNA yields

      Metagenomic sequencing

      Coverage analysis

      Genomic assembly and annotation

4. **Supplementary References**

1. Supplementary Figures

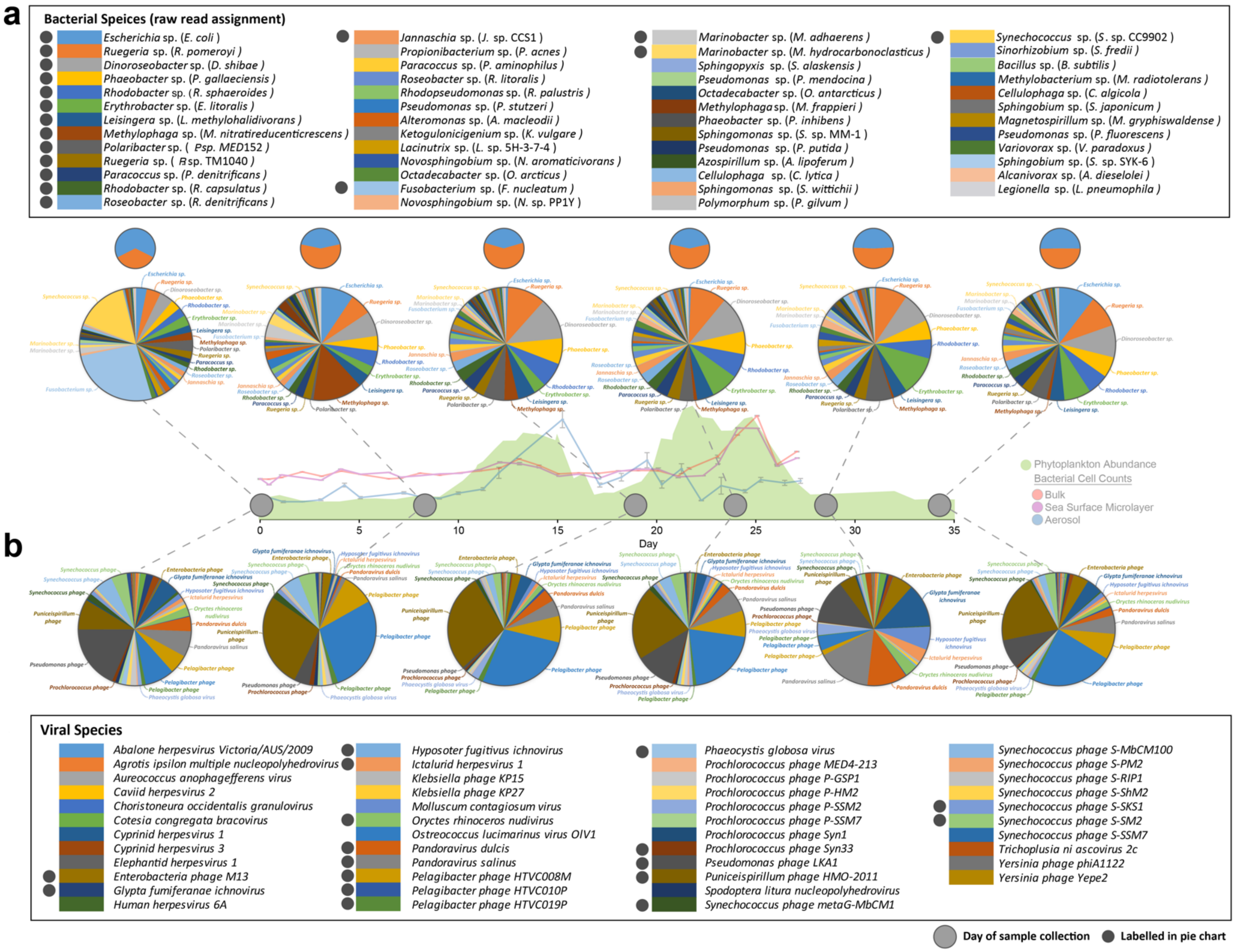

Supplementary Fig. 1 Bacterial and viral genome variation across the phytoplankton blooms. Bacteria (51 most abundant genomes), **a**, and viruses **b**, as determined by read based analysis on abundance trimmed data. Legends indicate species represented with dark gray circles denoting those that are labelled in the chart. Light gray circles on middle plot indicate day of sampling. Middle Plot shows phytoplankton abundance and bacterial abundance as reported in Figure 1. **a**, Orange portion of small pie charts above larger species pie charts represents 51 abundant genomes during the experiment and blue represents the remainder of the population. Abundance determined by number of samples above 0.5% of population and by sum of fraction of samples across blooms. Expanded pie charts demonstrate the identity and abundance of these species. Identities derived from bulk 0.2–3 μm samples. **b,** Virus species from 0.025–0.2 μm bulk samples. Error bars indicate s.d. and estimated error in aerosol measurements.

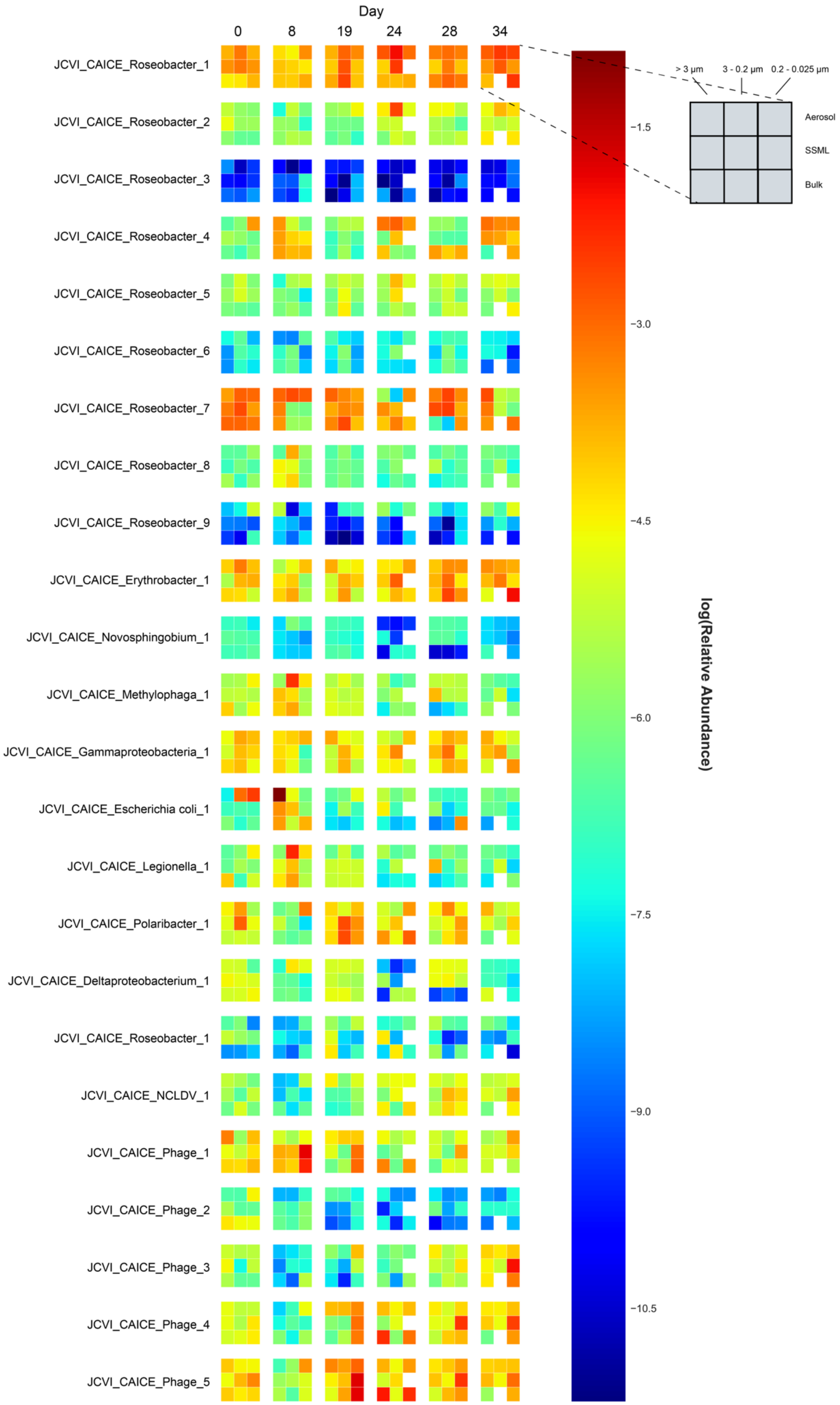

Supplementary Fig. 2 Abundance of draft genomes**.** 18 bacterial and 6 viral draft genomes determined by assembly based methods. Heatmap indicates fractional abundance of species in the sample. Day of sample is indicated on top columns. For each 9-square group size fraction is indicated from left to right: >3 µm, 0.02 – 3 µm, and 0.025 – 0.2 µm, and top to bottom: Aerosol, SSML, and bulk water compartments. Schematic in upper right corner indicates this sample arrangement. Blanks indicate samples below threshold limits.

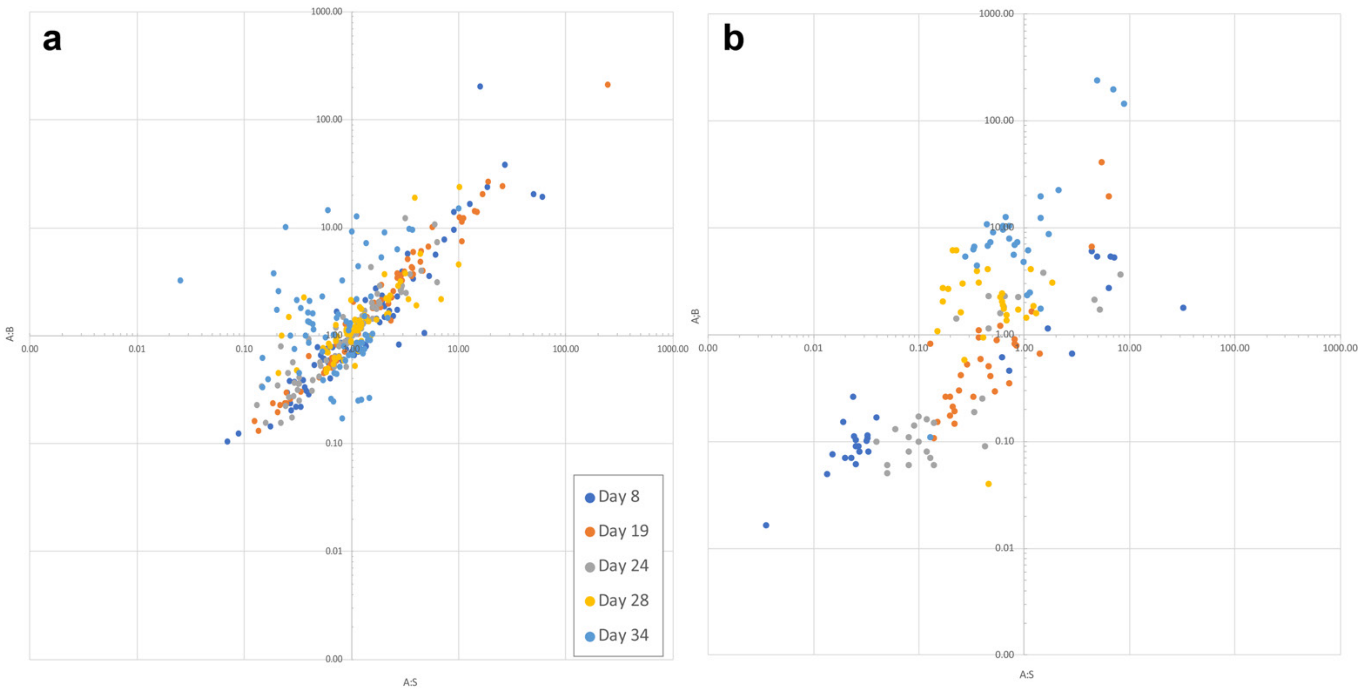

Supplementary Fig. 3 Genome spatial coverage trimmed bacterial and viral populations aerosolization distribution. The ratio of fraction of genomes in aerosols to the fraction in bulk plotted against ratio of fraction of genomes in aerosols to those in sea surface is shown for bacteria, **a**, and viruses, **b**, in genome spatial coverage trimmed data sets on select days. The upper right quadrants of **a** and **b** indicate genomes enriched in aerosol relative to both bulk and SSML (A:B and A:S > 1); the lower left quadrants indicates genomes that are primarily waterborne (A:B and A:S < 1). The upper left quadrants indicates genomes enriched relative to bulk but not SSML (A:B > 1, A:S < 1), and the lower right indicates genomes enriched in aerosol relative to the surface but not bulk (A:B < 1, A:S > 1). The data sets represent **a**, 76 bacterial genomes, and **b**, 30 viral genomes identified by read-based taxonomic assignments trimmed of species with low genome spatial coverage.

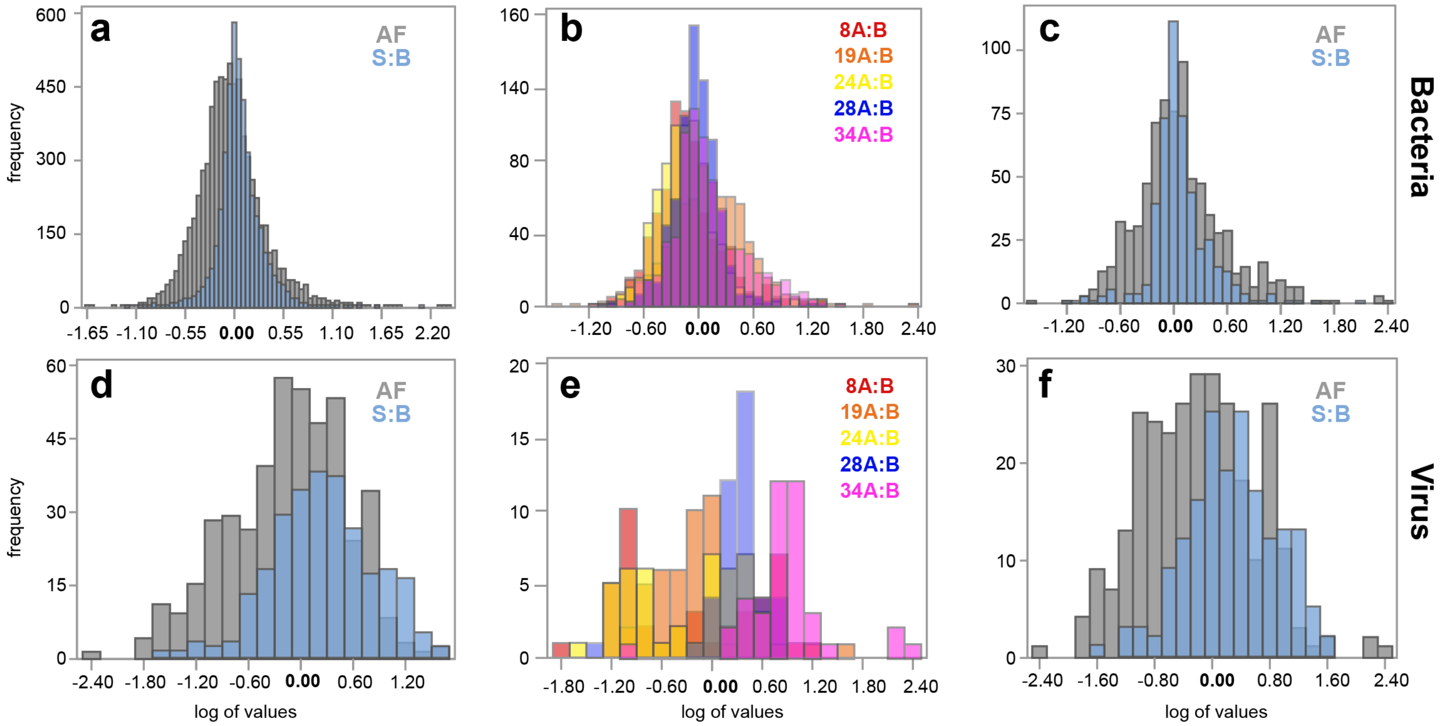

Supplementary Fig. 4 Population distribution of aerosolization and surface enrichment data sets. Histograms of all aerosolization factors (AFs) and surface enrichment factors (S:B) for bacteria, **a–c**, and viruses, **d–f** given by log values. **a, d** represent pooled AF and S:B values for abundance trimmed but not coverage trimmed data. **b, e** demonstrate day resolved variance in A:B distributions of these partially trimmed data sets. **c, f** show the pooled AF and S:B values for the fully trimmed data sets.

			Day	8		19		24		28		34			0	8	19	24	28	34		
Order	Class	Phylum	Species / AF	A:B	A:S	A:B	A:S	A:B	A:S	A:B	A:S	A:B	A:S	Avg AF	S:B	S:B	S:B	S:B	S:B	S:B	Avg S:B	Abundance
Fusobacteriales	Fusobacteriia	Fusobacteria	*Fusobacterium nucleatum*	1.65	0.73	1.99	2.23	0.34	0.15	1.48	0.26	1.27	0.43	1.05	0.09	2.26	0.89	2.31	5.66	2.92	2.36	
Bacteroidales	Bacteroidia	Bacteroidetes	*Bacteroides vulgatus*	0.60	0.85	0.82	0.73	0.54	0.69	0.59	0.84	0.33	0.15	0.61	1.48	0.71	1.12	0.77	0.70	2.23	1.17	
Cytophagales	Cytophagia	Bacteroidetes	*Cyclobacterium marinum*	0.22	0.33	0.13	0.13	0.64	0.76	2.11	2.23	0.39	0.54	0.75	2.36	0.66	0.97	0.84	0.95	0.72	1.08	
Flavobacteriales	Flavobacteriia	Bacteroidetes	*Nonlabens dokdonensis*	0.14	0.17	0.26	0.27	0.36	0.32	1.00	1.51	0.24	0.68	0.50	1.95	0.82	0.96	1.11	0.66	0.36	0.98	
Flavobacteriales	Flavobacteriia	Bacteroidetes	*Croceibacter atlanticus*	0.29	0.40	0.24	0.25	0.38	0.44	0.72	1.15	0.26	1.46	0.56	1.60	0.72	0.96	0.88	0.62	0.18	0.83	
Flavobacteriales	Flavobacteriia	Bacteroidetes	*Flavobacterium indicum*	0.20	0.27	0.20	0.20	0.22	0.24	0.89	1.24	0.25	1.16	0.49	2.32	0.74	0.95	0.91	0.72	0.21	0.98	
Flavobacteriales	Flavobacteriia	Bacteroidetes	*Zunongwangia profunda*	0.22	0.30	0.30	0.34	0.31	0.32	0.86	1.14	0.26	0.64	0.47	2.48	0.72	0.89	0.99	0.75	0.41	1.04	
Flavobacteriales	Flavobacteriia	Bacteroidetes	*Cellulophaga algicola*	0.24	0.27	0.23	0.21	0.27	0.26	0.81	1.12	0.25	1.24	0.49	1.83	0.88	1.06	1.05	0.72	0.20	0.96	
Flavobacteriales	Flavobacteriia	Bacteroidetes	*Cellulophaga lytica*	0.31	0.38	0.24	0.24	0.27	0.29	0.78	1.14	0.17	0.82	0.46	1.92	0.81	0.99	0.95	0.69	0.21	0.93	
Prochlorales		Cyanobacteria	*Prochlorococcus marinus*	0.39	0.35	2.13	1.89	0.51	0.89	2.14	0.98	2.17	1.12	1.26	0.14	1.09	1.13	0.58	2.17	1.94	1.17	
Lactobacillales	Bacilli	Firmicutes	*Streptococcus thermophilus*	20.5	50.3	10.1	5.66	4.29	1.51	1.56	2.14	10.2	0.24	10.7	0.06	0.41	1.79	2.85	0.73	41.9	7.96	
Lactobacillales	Bacilli	Firmicutes	*Streptococcus suis*	0.12	0.09	14.1	14.2	7.29	6.33	2.19	3.39	12.8	1.11	6.15	1.19	1.39	1.00	1.15	0.65	11.5	2.81	
Bacillales	Bacilli	Firmicutes	*Staphylococcus epidermidis*	7.74	7.40	5.11	3.36	2.87	1.85	1.02	0.97	2.12	0.31	3.27	1.04	1.05	1.52	1.56	1.05	6.87	2.18	
Bacillales	Bacilli	Firmicutes	*Bacillus cereus*	204	16.0	3.95	4.64	0.52	0.69	4.55	9.97	9.57	3.72	25.7	4.80	12.7	0.85	0.76	0.46	2.57	3.70	
Nakamurellales	Actinobacteria	Actinobacteria	*Nakamurella multipartita*	0.86	0.79	3.56	3.61	2.52	2.91	2.90	2.74	6.33	2.67	2.89	2.88	1.09	0.99	0.87	1.06	2.37	1.54	
Propionibacteriales	Actinobacteria	Actinobacteria	*Cutibacterium acnes*	38.5	26.9	20.5	16.8	12.2	3.15	23.6	10.2	14.4	0.60	16.7	2.38	1.43	1.22	3.89	2.31	23.9	5.86	
Micrococcales	Actinobacteria	Actinobacteria	*Rothia mucilaginosa*	1.05	4.78	212	246	3.96	4.45	19.0	3.87	3.26	0.03	49.8	0.53	0.22	0.86	0.89	4.90	129	22.70	
Micrococcales	Actinobacteria	Actinobacteria	*Micrococcus luteus*	13.9	9.00	26.4	18.7	10.8	6.03	1.40	1.71	9.20	1.01	9.82	0.48	1.54	1.41	1.79	0.82	9.07	2.52	
Geodermatophilales	Actinobacteria	Actinobacteria	*Geodermatophilus obscurus*	2.22	2.23	12.5	10.1	1.38	1.37	1.12	1.05	1.73	0.20	3.39	0.73	1.00	1.23	1.01	1.07	8.60	2.27	
Geodermatophilales	Actinobacteria	Actinobacteria	*Blastococcus saxobsidens*	2.33	1.93	24.2	25.5	2.47	3.23	1.55	1.32	2.58	0.21	6.53	1.76	1.21	0.95	0.76	1.18	12.5	3.05	
Corynebacteriales	Actinobacteria	Actinobacteria	*Gordonia polyisoprenivorans*	1.33	1.85	1.38	2.33	1.43	1.58	1.41	1.08	1.53	1.33	1.52	1.10	0.72	0.59	0.90	1.30	1.15	0.96	
Corynebacteriales	Actinobacteria	Actinobacteria	*Rhodococcus opacus*	2.14	1.35	2.60	2.47	2.00	1.54	1.25	0.71	4.41	1.15	1.96	2.23	1.59	1.05	1.30	1.76	3.83	1.96	
Corynebacteriales	Actinobacteria	Actinobacteria	*Rhodococcus erythropolis*	5.68	3.35	2.26	2.36	2.04	1.81	3.70	2.03	14.9	10.0	4.82	3.16	1.70	0.96	1.12	1.82	1.49	1.71	
Corynebacteriales	Actinobacteria	Actinobacteria	*Mycobacterium chubuense*	3.54	5.35	7.41	10.8	2.91	2.95	0.49	0.58	9.05	2.02	4.51	1.67	0.66	0.69	0.99	0.83	4.47	1.55	
Corynebacteriales	Actinobacteria	Actinobacteria	*Mycobacterium avium*	5.56	6.05	11.2	10.7	3.14	6.25	0.53	1.08	5.27	1.90	5.17	0.92	0.92	1.05	0.50	0.49	2.78	1.11	
Corynebacteriales	Actinobacteria	Actinobacteria	*Corynebacterium aurimucosum*	9.49	9.00	14.0	14.8	2.41	1.74	2.19	6.85	3.73	0.19	6.44	2.64	1.05	0.95	1.38	0.32	19.9	4.38	
Corynebacteriales	Actinobacteria	Actinobacteria	*Corynebacterium kroppenstedtii*	0.79	0.49	0.29	0.25	0.17	0.28	0.63	0.69	0.40	0.17	0.41	1.47	1.62	1.18	0.62	0.91	2.39	1.36	
Campylobacterales	Epsilonproteobacteria	Proteobacteria	*Sulfurimonas denitrificans*	0.10	0.07	2.05	1.04	0.15	0.16	1.90	4.05	0.86	1.11	1.15	0.78	1.50	1.97	0.98	0.47	0.78	1.08	
Burkholderiales	Betaproteobacteria	Proteobacteria	*Variovorax paradoxus*	1.14	1.13	2.00	1.77	1.04	1.12	1.06	0.99	1.46	0.72	1.24	1.69	1.01	1.13	0.93	1.07	2.02	1.31	
Burkholderiales	Betaproteobacteria	Proteobacteria	*Paraburkholderia xenovorans*	23.8	18.5	3.70	3.76	0.92	0.40	1.09	1.00	9.68	3.49	6.63	2.57	1.29	0.98	2.30	1.09	2.78	1.83	
Burkholderiales	Betaproteobacteria	Proteobacteria	*Ralstonia pickettii*	0.89	0.89	1.49	1.39	0.83	0.91	0.91	0.72	1.11	0.93	1.01	1.70	1.01	1.07	0.91	1.26	1.19	1.19	
Legionellales	Gammaproteobacteria	Proteobacteria	*Legionella pneumophila*	1.74	2.69	0.84	0.74	0.36	0.29	1.03	0.91	0.75	0.29	0.96	1.24	0.64	1.14	1.24	1.12	2.55	1.32	
Enterobacteriales	Gammaproteobacteria	Proteobacteria	*Serratia marcescens*	0.55	0.61	1.78	1.61	0.93	0.94	1.10	0.89	0.96	0.57	0.99	0.80	0.90	1.11	0.99	1.23	1.69	1.12	
Enterobacteriales	Gammaproteobacteria	Proteobacteria	*Rahnella aquatilis*	0.76	0.60	1.80	1.57	1.48	0.84	0.48	0.31	1.23	0.95	1.00	1.25	1.28	1.15	1.78	1.55	1.29	1.38	
Oceanospirillales	Gammaproteobacteria	Proteobacteria	*Thalassolituus oleivorans*	0.29	0.25	2.94	1.90	0.58	0.58	1.82	1.19	2.29	0.53	1.24	1.08	1.17	1.55	1.00	1.53	4.30	1.77	
Oceanospirillales	Gammaproteobacteria	Proteobacteria	*Alcanivorax dieselolei*	2.07	1.94	3.42	2.65	1.13	1.13	0.83	0.61	1.56	0.75	1.61	1.45	1.07	1.29	1.00	1.36	2.09	1.38	
Xanthomonadales	Gammaproteobacteria	Proteobacteria	*Rhodanobacter denitrificans*	16.4	12.6	12.2	11.1	0.73	0.57	1.36	1.44	1.01	0.68	5.82	1.18	1.30	1.10	1.28	0.94	1.48	1.21	
Xanthomonadales	Gammaproteobacteria	Proteobacteria	*Stenotrophomonas maltophilia*	1.88	2.04	4.24	3.73	0.89	1.00	0.98	0.74	1.00	0.37	1.69	1.58	0.92	1.14	0.89	1.33	2.69	1.43	
Thiotrichales	Gammaproteobacteria	Proteobacteria	*Methylophaga nitratireducenticrescens*	0.83	2.76	1.03	1.16	1.18	0.71	5.74	4.42	0.62	0.15	1.86	3.38	0.30	0.89	1.65	1.30	4.24	1.96	
Thiotrichales	Gammaproteobacteria	Proteobacteria	*Methylophaga frappieri*	1.53	2.44	1.39	1.37	0.45	0.26	3.80	3.16	1.00	0.27	1.57	3.40	0.62	1.02	1.75	1.20	3.75	1.96	
Alteromonadales	Gammaproteobacteria	Proteobacteria	*Idiomarina loihiensis*	0.61	0.56	0.64	0.40	0.34	0.20	0.76	0.54	0.70	0.54	0.53	1.04	1.10	1.60	1.68	1.40	1.31	1.36	
Alteromonadales	Gammaproteobacteria	Proteobacteria	*Alteromonas macleodii*	1.37	1.15	6.04	4.49	0.56	0.28	1.37	1.19	2.09	0.40	1.89	0.98	1.19	1.34	1.99	1.15	5.20	1.97	
Alteromonadales	Gammaproteobacteria	Proteobacteria	*Marinobacter hydrocarbonoclasticus*	0.33	0.37	3.90	3.12	0.52	0.51	1.39	1.08	1.35	0.39	1.30	1.44	0.90	1.25	1.01	1.28	3.47	1.56	
Alteromonadales	Gammaproteobacteria	Proteobacteria	*Marinobacter adhaerens*	0.40	0.63	4.80	4.41	0.56	0.51	1.76	1.23	1.81	0.34	1.64	0.86	0.64	1.09	1.11	1.43	5.39	1.75	
Pseudomonadales	Gammaproteobacteria	Proteobacteria	*Moraxella catarrhalis*	0.37	0.29	0.16	0.13	0.40	0.32	1.01	0.69	0.45	0.33	0.42	0.63	1.25	1.27	1.25	1.45	1.38	1.21	
Pseudomonadales	Gammaproteobacteria	Proteobacteria	*Acinetobacter calcoaceticus*	19.2	60.4	3.22	2.99	2.71	2.92	1.61	2.23	1.36	0.89	9.76	1.01	0.32	1.08	0.93	0.72	1.53	0.93	
Pseudomonadales	Gammaproteobacteria	Proteobacteria	*Pseudomonas fluorescens*	1.84	1.61	3.75	2.69	0.79	0.76	0.89	0.71	1.61	0.43	1.51	1.52	1.15	1.40	1.03	1.26	3.73	1.68	
Pseudomonadales	Gammaproteobacteria	Proteobacteria	*Pseudomonas mendocina*	0.53	0.45	6.66	5.25	2.61	2.87	2.33	2.29	7.20	1.37	3.16	0.98	1.19	1.27	0.91	1.02	5.27	1.77	
Pseudomonadales	Gammaproteobacteria	Proteobacteria	*Pseudomonas stutzeri*	0.75	0.89	4.32	3.63	1.83	1.56	1.13	0.89	1.63	0.39	1.70	0.89	0.84	1.19	1.17	1.27	4.14	1.58	
Pseudomonadales	Gammaproteobacteria	Proteobacteria	*Pseudomonas fulva*	0.66	0.51	2.01	1.62	0.85	0.77	0.70	0.58	1.29	0.97	1.00	1.64	1.28	1.24	1.11	1.21	1.33	1.30	
Pseudomonadales	Gammaproteobacteria	Proteobacteria	*Pseudomonas putida*	0.59	0.61	3.60	2.92	0.64	0.71	0.80	0.63	1.32	0.41	1.22	1.14	0.96	1.23	0.91	1.26	3.25	1.46	
	Alphaproteobacteria	Proteobacteria	*Polymorphum gilvum*	0.59	0.71	0.62	0.65	1.15	1.24	1.45	1.36	0.79	0.84	0.94	3.31	0.83	0.95	0.93	1.07	0.94	1.34	
	Alphaproteobacteria	Proteobacteria	*Candidatus Puniceispirillum marinum*	0.59	0.54	0.83	0.83	1.00	1.03	0.69	0.60	1.46	2.18	0.97	0.15	1.10	1.00	0.97	1.15	0.67	0.84	
Rhizobiales	Alphaproteobacteria	Proteobacteria	*Bradyrhizobium diazoefficiens*	3.33	3.78	1.84	1.86	1.13	1.09	3.18	2.88	2.29	1.51	2.29	3.06	0.88	0.99	1.04	1.11	1.51	1.43	
Rhizobiales	Alphaproteobacteria	Proteobacteria	*Methylobacterium radiotolerans*	3.93	2.95	7.32	6.32	3.67	3.53	2.22	2.09	3.21	0.82	3.61	2.43	1.33	1.16	1.04	1.06	3.92	1.82	
Pelagibacterales	Alphaproteobacteria	Proteobacteria	*Candidatus Pelagibacter ubique*	2.73	1.67	5.96	3.77	0.23	0.13	1.00	0.22	1.13	0.44	1.73	0.10	1.63	1.58	1.72	4.50	2.56	2.01	
	Alphaproteobacteria	Proteobacteria	*alpha proteobacterium HIMB59*	0.38	0.26	0.24	0.18	0.36	0.32	0.45	0.21	0.52	0.77	0.37	0.10	1.43	1.28	1.11	2.13	0.67	1.12	
	Alphaproteobacteria	Proteobacteria	*alpha proteobacterium HIMB5*	0.78	0.67	1.28	0.88	0.79	0.22	2.25	0.36	0.86	0.40	0.85	0.11	1.16	1.45	3.60	6.29	2.14	2.46	
Sphingomonadales	Alphaproteobacteria	Proteobacteria	*Erythrobacter litoralis*	0.59	0.82	0.41	0.50	0.16	0.22	0.46	0.58	0.44	0.73	0.49	1.79	0.71	0.82	0.71	0.78	0.61	0.90	
Sphingomonadales	Alphaproteobacteria	Proteobacteria	*Sphingomonas wittichii*	1.43	1.72	0.70	0.86	0.51	0.69	0.60	0.71	0.66	0.94	0.88	2.55	0.83	0.82	0.73	0.85	0.70	1.08	
Sphingomonadales	Alphaproteobacteria	Proteobacteria	*Sphingobium japonicum*	1.68	2.24	0.48	0.57	0.41	0.50	0.50	0.60	0.58	0.88	0.84	2.08	0.75	0.85	0.83	0.83	0.67	1.00	
Sphingomonadales	Alphaproteobacteria	Proteobacteria	*Novosphingobium aromaticivorans*	2.29	2.65	0.56	0.65	0.25	0.33	0.54	0.67	0.45	0.69	0.91	1.99	0.86	0.86	0.77	0.80	0.65	0.99	
Sphingomonadales	Alphaproteobacteria	Proteobacteria	*Sphingopyxis alaskensis*	1.48	2.03	0.59	0.72	0.31	0.43	0.63	0.76	0.66	1.02	0.86	2.00	0.73	0.82	0.71	0.82	0.64	0.96	
Rhodobacterales	Alphaproteobacteria	Proteobacteria	*Leisingera methylohalidivorans*	0.76	1.20	0.50	0.61	1.76	1.70	1.25	1.19	1.00	1.38	1.14	3.08	0.63	0.82	1.04	1.05	0.72	1.22	
Rhodobacterales	Alphaproteobacteria	Proteobacteria	*Ketogulonicigenium vulgare*	0.68	1.01	0.52	0.68	1.23	1.14	1.11	1.09	0.73	1.04	0.92	2.33	0.67	0.77	1.08	1.02	0.70	1.09	
Rhodobacterales	Alphaproteobacteria	Proteobacteria	*Ruegeria pomeroyi*	0.83	1.34	0.51	0.62	1.83	1.68	1.26	1.24	1.04	1.58	1.19	3.37	0.61	0.83	1.09	1.02	0.66	1.26	
Rhodobacterales	Alphaproteobacteria	Proteobacteria	*Dinoroseobacter shibae*	0.70	1.26	0.50	0.66	1.51	1.25	1.21	1.08	0.86	1.26	1.03	3.83	0.56	0.76	1.21	1.12	0.68	1.36	
Rhodobacterales	Alphaproteobacteria	Proteobacteria	*Paracoccus denitrificans*	0.69	1.07	0.60	0.72	1.28	1.29	1.18	1.15	0.79	1.12	0.99	3.85	0.65	0.83	1.00	1.03	0.71	1.34	
Rhodobacterales	Alphaproteobacteria	Proteobacteria	*Phaeobacter gallaeciensis*	0.80	1.37	0.52	0.68	1.68	1.33	1.29	1.19	0.91	1.41	1.12	3.41	0.59	0.76	1.26	1.09	0.65	1.29	
Rhodobacterales	Alphaproteobacteria	Proteobacteria	*Phaeobacter inhibens*	0.76	1.12	0.59	0.69	1.96	1.82	1.18	1.22	1.01	1.36	1.17	2.40	0.68	0.85	1.07	0.96	0.75	1.12	
Rhodobacterales	Alphaproteobacteria	Proteobacteria	*Rhodobacter sphaeroides*	0.66	1.06	0.53	0.64	1.45	1.37	1.17	1.11	0.78	1.17	1.00	3.62	0.62	0.84	1.06	1.06	0.67	1.31	
Rhodobacterales	Alphaproteobacteria	Proteobacteria	*Rhodobacter capsulatus*	0.70	1.07	0.45	0.55	1.33	1.21	1.16	1.09	0.86	1.21	0.96	3.15	0.65	0.81	1.10	1.06	0.71	1.25	
Rhodobacterales	Alphaproteobacteria	Proteobacteria	*Roseobacter litoralis*	0.71	1.15	0.55	0.67	1.41	1.42	1.12	1.15	0.73	1.18	1.01	1.93	0.62	0.83	0.99	0.97	0.62	0.99	
Rhodobacterales	Alphaproteobacteria	Proteobacteria	*Roseobacter denitrificans*	0.69	1.03	0.45	0.55	1.36	1.34	1.30	1.23	0.91	1.49	1.03	2.75	0.67	0.81	1.01	1.06	0.61	1.15	
Rhodobacterales	Alphaproteobacteria	Proteobacteria	*Octadecabacter antarcticus*	0.74	1.22	0.45	0.56	0.87	0.86	0.90	0.99	0.75	1.32	0.86	1.42	0.60	0.80	1.01	0.91	0.57	0.88	
Rhodobacterales	Alphaproteobacteria	Proteobacteria	*Octadecabacter arcticus*	0.71	1.00	0.53	0.65	0.85	1.00	0.88	0.98	0.68	1.29	0.86	1.44	0.71	0.81	0.85	0.90	0.53	0.87	

Aerosolization — Surface Enhancement

> 5.0	1.5 - 5.0	1.0 - 1.5	0.67 - 1.0	0.2 - 0.67	< 0.2

Enriched ← Neutral Transfer → Diminished

Total fraction / Samples > 0.5%

Supplementary Fig. 5 Bacterial aerosolization and surface enrichment. Taxonomy, aerosolization factors, and surface enrichment of bacterial genomes of spatial coverage and abundance trimmed data. Phylum, class, and orders are color coded to reflect species relationships. Genome annotation by read-based analysis, Heat map column labels indicate day and measurement. Aerosolization factor is expressed as the fraction of genomes in aerosol to bulk or surface (A:B or A:S). Surface enrichment is the fraction in SSML to the fraction in the bulk (S:B). Heat map values indicate ratio values (bottom legend). Species abundance is reported as the sum of fraction of the population across the experiment and as the number of samples above 0.5% of the population.

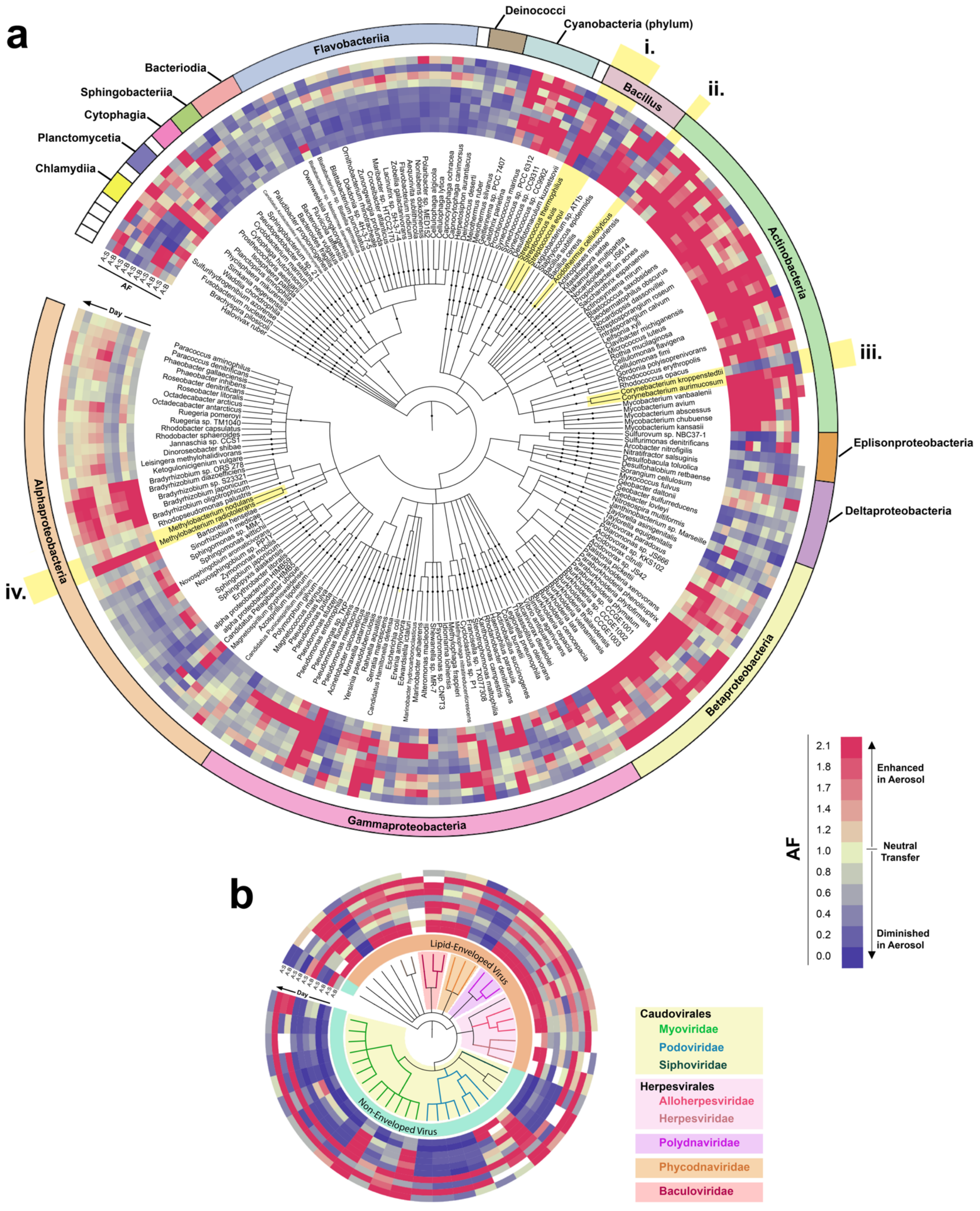

Supplementary Fig. 6 The genetic basis of bacterial and viral aerosolization in the abundance trimmed data set. Genome-based phylogenetic trees based on 197 bacterial genome sequences in **a** and 46 viral genomes in **b.** Sequences are derived from read-centric analysis of above 0.1% of the population but not trimmed of genomes with low spatial coverage. The bacteria are a subset of this data that contains 700 bacterial genomes total. The heatmaps display aerosolization factor (AF), A:B and A:S, on different days. AF intensity specified by the legend: Blue = Diminished aerosolization; Yellow = Neutral aerosolization; Red = Enhanced aerosolization. **a**, Bacteria class is indicated on outer ring to further denote species and aerosolization relationships. Yellow highlights, i –iv indicate closely related species with differing aerosolization patterns. **b**, Line colors denote viral family and shading indicates order as shown by legend. Additionally, labels on the inner ring specify lipid-enveloped or non-enveloped classifications. Blanks indicate samples below threshold limits.

Viral Draft genomes (0.025 µm fraction)	Day 8 A:B	8 A:S	19 A:B	19 A:S	24 A:B	24 A:S	28 A:B	28 A:S	34 A:B	34 A:S	Avg AF	0 S:B	8 S:B	19 S:B	24 S:B	28 S:B	34 S:B	Avg S:B	Abundance
JCVI_CAICE_NCLDV_1	3.18	11.2	2.82	2.11	0.60	0.83	0.85	0.60	0.68	0.28	2.3	2.60	0.28	1.33	0.72	1.41	2.42	1.5	
JCVI_CAICE_Phage_1	0.09	0.06	0.34	0.37	0.23	0.18	1.29	0.34	5.94	1.76	1.1	0.61	1.38	0.93	1.29	3.84	3.38	1.9	
JCVI_CAICE_Phage_2	0.35	0.35	0.82	0.53	0.40	0.31	1.40	0.26	2.17	0.80	0.7	0.19	1.01	1.54	1.29	5.32	2.71	2.0	
JCVI_CAICE_Phage_3	0.20	1.52	19.8	3.81	0.55	0.78	1.76	0.53	0.43	0.14	3.0	3.26	0.13	5.20	0.71	3.32	3.14	2.6	
JCVI_CAICE_Phage_4	2.48	10.7	0.84	0.76	0.44	0.20	0.72	0.17	0.63	0.24	1.7	1.06	0.23	1.11	2.15	4.30	2.60	1.9	
JCVI_CAICE_Phage_5	4.00	4.11	0.33	0.31	0.30	0.23	0.94	0.33	0.78	0.38	1.2	2.35	0.97	1.05	1.32	2.90	2.07	1.8	

Bacterial draft genomes (0.02 µm fraction)	8A:B	8A:S	19A:B	19A:S	24A:B	24A:S	28A:B	28A:S	34A:B	34A:S	Avg AF	0S:B	8S:B	19S:B	24S:B	28S:B	34S:B	Avg S:B	Abundance
JCVI_CAICE_Erythrobacter_1	0.36	0.53	0.31	0.38	0.19	0.23	0.45	0.65	0.31	0.67	0.4	1.56	0.69	0.82	0.82	0.69	0.46	0.8	
JCVI_CAICE_Novosphingobium_1	6.39	6.73	0.74	1.38	0.08	0.60	60.4	0.92	0.31	0.96	7.9	1.23	0.95	0.53	0.13	65.6	0.33	11.5	
JCVI_CAICE_Methylophaga_1	3.50	6.53	1.26	1.70	0.31	0.21	12.6	1.20	0.28	0.23	2.8	1.56	0.54	0.74	1.52	10.4	1.21	2.7	
JCVI_CAICE_Gammaproteobacteria_1	1.92	1.46	0.45	0.45	0.13	0.12	1.05	0.54	0.29	0.37	0.7	1.02	1.32	0.99	1.06	1.94	0.78	1.2	
JCVI_CAICE_Escherichia coli_1	1.55	0.47	5.69	0.58	3.47	1.79	3.27	2.43	0.25	0.88	2.0	1.53	3.29	9.89	1.93	1.35	0.28	3.0	
JCVI_CAICE_Legionella_1	3.83	5.49	0.97	0.86	1.17	0.28	4.94	3.03	1.1	0.23	2.2	3.87	0.70	1.12	4.17	1.63	4.80	2.7	
JCVI_CAICE_Polaribacter_1	1.82	1.49	0.11	0.11	0.40	0.23	2.86	3.55	0.38	1.43	1.2	7.99	1.22	1.00	1.73	0.81	0.27	2.2	
JCVI_CAICE_Deltaproteobacteria_1	5.84	7.89	0.81	1.65	0.02	0.30	58.8	0.82	0.13	0.91	7.7	1.15	0.74	0.49	0.06	71.3	0.14	12.3	
JCVI_CAICE_Diatom_1	2.19	0.66	4.12	3.37	0.83	3.51	6.78	25.1	6.16	8.23	6.1	15.2	3.32	1.22	0.24	0.27	0.75	3.5	
JCVI_CAICE_Roseobacter_1	0.63	0.69	0.68	0.81	1.97	1.67	0.90	1.17	1.10	1.41	1.1	3.32	0.91	0.85	1.18	0.77	0.78	1.3	
JCVI_CAICE_Roseovarius_1	1.21	1.81	1.75	1.89	14.3	11.5	2.41	2.93	4.54	4.62	4.7	1.94	0.67	0.92	1.25	0.82	0.98	1.1	
JCVI_CAICE_Roseobacter_fragments_1	0.14	0.08	1.79	6.04	0.49	1.01	0.42	1.91	1.53	0.77	1.4	0.31	1.72	0.30	0.49	0.22	2.00	0.8	
JCVI_CAICE_Roseobacter_fragments_2	0.32	0.42	1.20	1.66	12.6	2.82	0.21	2.42	8.36	1.34	3.1	2.78	0.76	0.72	4.49	0.09	6.22	2.5	
JCVI_CAICE_Roseobacter_fragments_3	1.35	2.13	0.55	0.67	1.84	1.48	1.28	1.31	0.69	1.35	1.3	3.89	0.63	0.82	1.24	0.97	0.51	1.3	
JCVI_CAICE_Roseobacter_fragments_4	0.09	0.08	0.30	0.20	0.29	0.17	0.53	0.49	0.36	0.93	0.3	1.57	1.12	1.52	1.70	1.07	0.38	1.2	
JCVI_CAICE_Roseobacter_fragments_5	20.2	27.7	0.48	0.98	0.02	0.02	198	0.92	0.08	1.01	25.0	1.30	0.73	0.50	1.35	215	0.08	36.4	
JCVI_CAICE_Roseobacter_fragments_6	1.53	2.78	1.14	1.32	1.52	1.02	2.40	2.69	0.82	0.43	1.6	2.37	0.55	0.87	1.49	0.89	1.91	1.3	
JCVI_CAICE_Roseobacter_fragments_7	0.19	0.08	134	21.9	18.6	17.5	5.75	33.2	15.9	1.46	24.9	5.08	2.27	6.12	1.06	0.17	10.9	4.3	
JCVI_CAICE_Roseobacter_frag_contam	0.01	0.01	7.68	43.2	2.92	5.91	0.19	0.35	35.3	1.54	9.7	0.50	0.96	0.18	0.49	0.54	23.0	4.3	

Aerosolization — Surface Enhancement

Legend: >5.0 | 1.5 - 5.0 | 1.0 - 1.5 | 0.67 - 1.0 | 0.2 - 0.67 | <0.2

Enriched — Neutral Transfer — Diminished

Total fraction | Samples > 0.5%

Supplementary Fig. 7 Draft genome bacterial and viral aerosolization and surface enrichment. Taxonomy, aerosolization factors, and surface enrichment of assembled draft genomes. Heat map column labels indicate day and measurement. Aerosolization factor is expressed as the fraction of species in aerosol to bulk or surface (A:B or A:S). Surface enrichment is the fraction in SSML to the fraction in the bulk (S:B). Heat map values indicate ratio values (bottom legend). Genome abundance is reported as the sum of fraction of the population across the experiment and as the number of samples above 0.5% of the population.

a

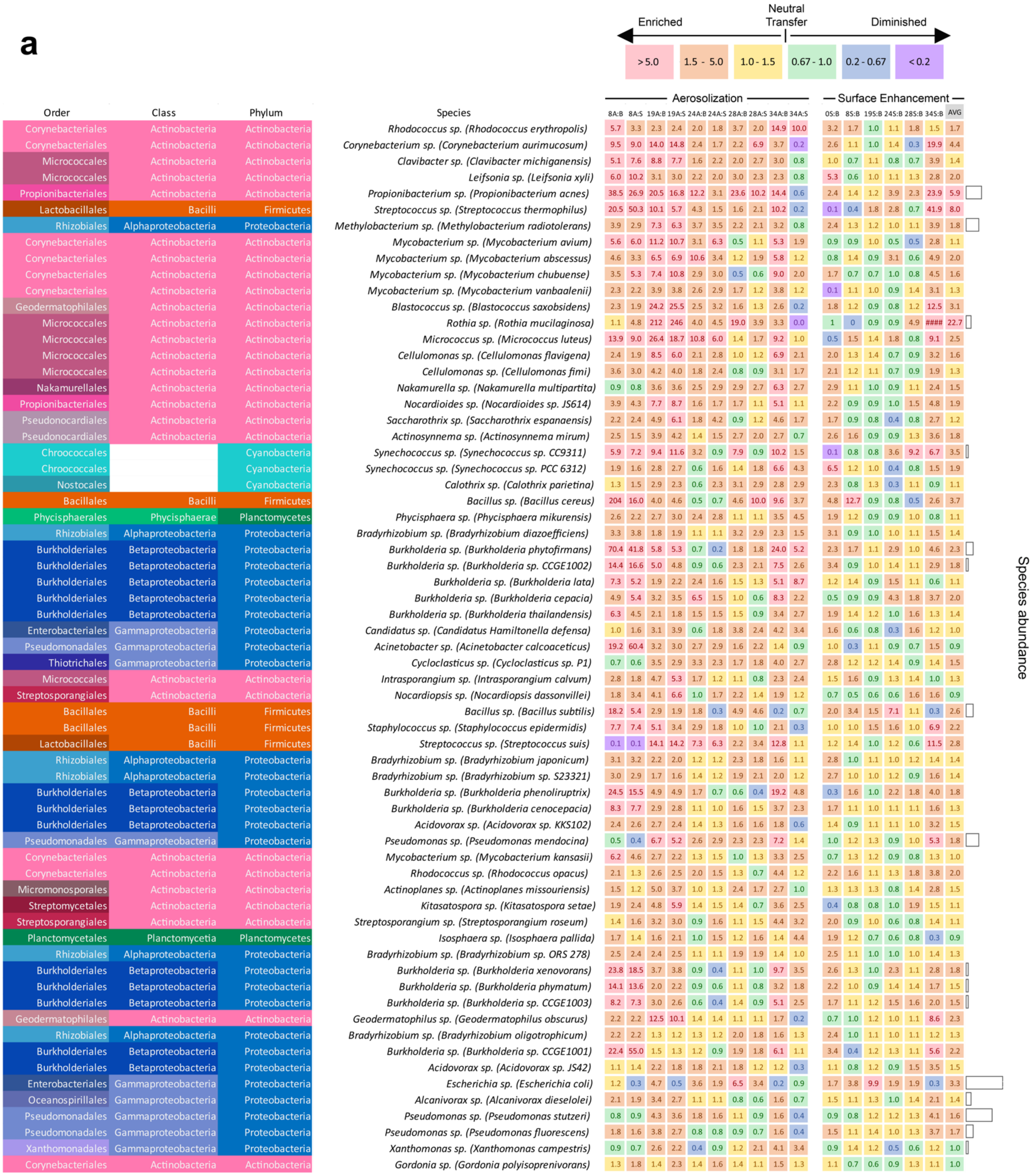

Supplementary Fig. 8 Bacterial aerosolization of abundance trimmed data set. Taxonomy, aerosolization factors, and surface enrichment of 197 bacterial genomes from abundance trimmed but not coverage trimmed data presented in three sets, **a**, **b**, **c**. Phylum, class, and orders are color coded to reflect genome relationships. Genome annotation by read-based analysis, exact assignment in parentheses. Heat map column labels indicate day and measurement as well as enrichment factor. Aerosolization factor is expressed as the fraction of a genome in aerosol to bulk or surface (A:B or A:S). Surface enrichment is the fraction in SSML to the fraction in the bulk (S:B). Heat map values indicate ratio values as given in legend. Bars on right indicate genome abundance (number of samples containing above 0.5% of the total population).

b

Enriched — Neutral Transfer — Diminished

> 5.0	1.5 - 5.0	1.0 - 1.5	0.67 - 1.0	0.2 - 0.67	< 0.2

Order	Class	Phylum	Species	Aerosolization 8A:B	8A:S	19A:B	19A:S	24A:B	24A:S	28A:B	28A:S	34A:B	34A:S	Surface Enhancement 0S:B	8S:B	19S:B	24S:B	28S:B	34S:B	AVG
Chroococcales		Cyanobacteria	*Synechococcus sp. (Synechococcus sp. CC9902)*	1.3	2.0	17.9	11.5	0.9	0.2	13.8	0.3	6.8	0.4	0.1	0.6	1.6	4.5	40.6	17.2	10.8
Prochlorales		Cyanobacteria	*Prochlorococcus sp. (Prochlorococcus marinus)*	0.4	0.4	2.1	1.9	0.5	0.9	2.1	1.0	2.2	1.1	0.1	1.1	1.1	0.6	2.2	1.9	1.2
	Alphaproteobacteria	Proteobacteria	*Candidatus sp. (Candidatus Pelagibacter ubique)*	2.7	1.7	6.0	3.8	0.2	0.1	1.0	0.2	1.1	0.4	0.1	1.6	1.6	1.7	4.5	2.6	2.0
Burkholderiales	Betaproteobacteria	Proteobacteria	*Burkholderia sp. (Burkholderia vietnamiensis)*	8.2	6.1	1.3	1.2	0.6	0.5	0.4	0.5	5.7	5.8	0.6	1.3	1.1	1.0	0.9	1.0	1.0
Xanthomonadales	Gammaproteobacteria	Proteobacteria	*Rhodanobacter sp. (Rhodanobacter denitrificans)*	16.4	12.6	12.2	11.1	0.7	0.6	1.4	1.4	1.0	0.7	1.2	1.3	1.1	1.3	0.9	1.5	1.2
Xanthomonadales	Gammaproteobacteria	Proteobacteria	*Stenotrophomonas sp. (Stenotrophomonas maltophilia)*	1.9	2.0	4.2	3.7	0.9	1.0	1.0	0.7	1.0	0.4	1.6	0.9	1.1	0.9	1.3	2.7	1.4
Rhodobacterales	Alphaproteobacteria	Proteobacteria	*Phaeobacter sp. (Phaeobacter inhibens)*	0.8	1.1	0.6	0.7	2.0	1.8	1.2	1.2	1.0	1.4	2.4	0.7	0.9	1.1	1.0	0.7	1.1
Rhodobacterales	Alphaproteobacteria	Proteobacteria	*Leisingera sp. (Leisingera methylohalidivorans)*	0.8	1.2	0.5	0.6	1.8	1.7	1.2	1.2	1.0	1.4	3.1	0.6	0.8	1.0	1.0	0.7	1.2
Rhodobacterales	Alphaproteobacteria	Proteobacteria	*Ruegeria sp. (Ruegeria sp. TM1040)*	0.9	1.5	0.6	0.7	1.5	1.5	1.1	1.0	1.0	1.3	2.3	0.6	0.8	1.0	1.1	0.8	1.1
Rhodobacterales	Alphaproteobacteria	Proteobacteria	*Ruegeria sp. (Ruegeria pomeroyi)*	0.8	1.3	0.5	0.6	1.8	1.7	1.3	1.2	1.0	1.6	3.4	0.6	0.8	1.1	1.0	0.7	1.3
Burkholderiales	Betaproteobacteria	Proteobacteria	*Acidovorax sp. (Acidovorax citrulli)*	1.1	1.0	1.9	2.0	1.4	1.3	1.2	1.2	2.3	0.9	1.4	1.1	1.0	1.1	0.9	2.6	1.4
Myxococcales	Deltaproteobacteria	Proteobacteria	*Sorangium sp. (Sorangium cellulosum)*	1.1	0.9	1.4	1.4	1.1	1.6	0.8	0.7	15.8	9.1	0.5	1.3	1.0	0.6	1.2	1.7	1.1
Rhizobiales	Alphaproteobacteria	Proteobacteria	*Bartonella sp. (Bartonella henselae)*	1.6	1.7	1.0	1.2	0.6	0.6	0.7	0.7	0.8	1.1	1.2	0.9	0.8	1.2	1.1	0.8	1.0
Legionellales	Gammaproteobacteria	Proteobacteria	*Legionella sp. (Legionella pneumophila)*	1.7	2.7	0.8	0.7	0.4	0.3	1.0	0.9	0.7	0.3	1.2	0.6	1.1	1.2	1.1	2.6	1.3
Alteromonadales	Gammaproteobacteria	Proteobacteria	*Alteromonas sp. (Alteromonas macleodii)*	1.4	1.2	6.0	4.5	0.6	0.3	1.4	1.2	2.1	0.4	1.0	1.2	1.3	2.0	1.1	5.2	2.0
Thiotrichales	Gammaproteobacteria	Proteobacteria	*Methylophaga sp. (Methylophaga nitratireducenticrescens)*	0.8	2.8	1.0	1.2	1.2	0.7	5.7	4.4	0.6	0.1	3.4	0.3	0.9	1.6	1.3	4.2	2.0
Vibrionales	Gammaproteobacteria	Proteobacteria	*Vibrio sp. (Vibrio sp. Ex25)*	0.3	0.2	1.8	1.3	1.6	0.9	0.8	0.7	1.6	0.9	0.3	1.1	1.4	1.7	1.1	1.8	1.2
Alteromonadales	Gammaproteobacteria	Proteobacteria	*Marinobacter sp. (Marinobacter adhaerens)*	0.4	0.6	4.8	4.4	0.6	0.5	1.8	1.2	1.8	0.3	0.9	0.6	1.1	1.1	1.4	5.4	1.8
Oceanospirillales	Gammaproteobacteria	Proteobacteria	*Thalassolituus sp. (Thalassolituus oleivorans)*	0.3	0.3	2.9	1.9	0.6	0.6	1.8	1.2	2.3	0.5	1.1	1.2	1.5	1.0	1.5	4.3	1.8
Thiotrichales	Gammaproteobacteria	Proteobacteria	*Methylophaga sp. (Methylophaga frappieri)*	1.5	2.4	1.4	1.4	0.4	0.3	3.8	3.2	1.0	0.3	3.4	0.6	1.0	1.7	1.2	3.8	2.0
Brachyspirales	Spirochaetia	Spirochaetes	*Brachyspira sp. (Brachyspira pilosicoli)*	0.5	0.4	2.7	2.0	0.5	0.5	1.0	0.6	5.5	1.8	0.1	1.4	1.3	1.0	1.7	3.1	1.4
Pseudomonadales	Gammaproteobacteria	Proteobacteria	*Pseudomonas sp. (Pseudomonas sp. TKP)*	1.1	1.0	11.5	11.4	0.5	0.7	0.7	0.6	1.0	0.5	1.1	1.1	1.0	0.8	1.2	1.9	1.2
Pseudomonadales	Gammaproteobacteria	Proteobacteria	*Pseudomonas sp. (Pseudomonas putida)*	0.6	0.6	3.6	2.9	0.6	0.7	0.8	0.6	1.3	0.4	1.1	1.0	1.2	0.9	1.3	3.3	1.5
Pseudomonadales	Gammaproteobacteria	Proteobacteria	*Pseudomonas sp. (Pseudomonas entomophila)*	0.6	0.7	2.1	2.1	0.7	0.8	0.7	0.6	1.1	0.8	2.7	0.9	1.0	0.8	1.1	1.4	1.3
Pseudomonadales	Gammaproteobacteria	Proteobacteria	*Pseudomonas sp. (Pseudomonas fulva)*	0.7	0.5	2.0	1.6	0.9	0.8	0.7	0.6	1.3	1.0	1.6	1.3	1.2	1.1	1.2	1.3	1.3
Rhodobacterales	Alphaproteobacteria	Proteobacteria	*Phaeobacter sp. (Phaeobacter gallaeciensis)*	0.8	1.4	0.5	0.7	1.7	1.3	1.3	1.2	0.9	1.4	3.4	0.6	0.8	1.3	1.1	0.6	1.3
Rhodobacterales	Alphaproteobacteria	Proteobacteria	*Dinoroseobacter sp. (Dinoroseobacter shibae)*	0.7	1.3	0.5	0.7	1.5	1.2	1.2	1.1	0.9	1.3	3.8	0.6	0.8	1.2	1.1	0.7	1.4
	Alphaproteobacteria	Proteobacteria	*Candidatus sp. (Candidatus Puniceispirillum marinum)*	0.6	0.5	0.8	0.8	1.0	1.0	0.7	0.6	1.5	2.2	0.2	1.1	1.0	1.0	1.1	0.7	0.8
	Alphaproteobacteria	Proteobacteria	*alpha sp. (alpha proteobacterium HIMB5)*	0.8	0.7	1.3	0.9	0.8	0.2	2.2	0.4	0.9	0.4	0.1	1.2	1.4	3.6	6.3	2.1	2.5
Burkholderiales	Betaproteobacteria	Proteobacteria	*Polaromonas sp. (Polaromonas sp. JS666)*	0.9	1.0	1.6	1.2	0.9	0.9	0.9	0.7	1.4	0.6	1.1	0.9	1.4	1.0	1.4	2.3	1.3
Myxococcales	Deltaproteobacteria	Proteobacteria	*Myxococcus sp. (Myxococcus fulvus)*	1.2	1.5	1.2	1.3	0.8	0.9	1.0	0.7	0.8	0.6	1.0	0.8	0.9	0.9	1.5	1.2	1.0
Planctomycetales	Planctomycetia	Planctomycetes	*Planctomyces sp. (Planctomyces limnophilus)*	0.6	1.2	1.1	1.4	0.6	1.2	0.6	0.6	1.7	0.8	1.4	0.5	0.8	0.5	1.0	2.0	1.0
Pasteurellales	Gammaproteobacteria	Proteobacteria	*Actinobacillus sp. (Actinobacillus succinogenes)*	1.4	2.1	0.5	0.5	0.3	0.4	2.5	1.3	0.5	0.5	1.6	0.7	1.0	0.7	1.8	1.0	1.1
Aquificales	Aquificae	Aquificae	*Sulfurihydrogenibium sp. (Sulfurihydrogenibium azorense)*	1.9	1.9	0.0	0.0	0.4	0.2	1.8	1.5	0.3	2.7	4.0	1.0	1.0	2.0	1.2	0.1	1.5
Sphingomonadales	Alphaproteobacteria	Proteobacteria	*Novosphingobium sp. (Novosphingobium aromaticivorans)*	2.3	2.7	0.6	0.7	0.3	0.3	0.5	0.7	0.4	0.7	2.0	0.9	0.9	0.8	0.8	0.7	1.0
Sphingomonadales	Alphaproteobacteria	Proteobacteria	*Sphingobium sp. (Sphingobium japonicum)*	1.7	2.2	0.5	0.6	0.4	0.5	0.5	0.6	0.6	0.9	2.1	0.8	0.8	0.8	0.8	0.7	1.0
Burkholderiales	Betaproteobacteria	Proteobacteria	*Variovorax sp. (Variovorax paradoxus)*	1.1	1.1	2.0	1.8	1.0	1.1	1.1	1.0	1.5	0.7	1.7	1.0	1.1	0.9	1.1	2.0	1.3
Burkholderiales	Betaproteobacteria	Proteobacteria	*Janthinobacterium sp. (Janthinobacterium sp. Marseille)*	0.8	0.6	2.6	2.0	0.5	0.4	0.7	0.6	1.2	0.6	0.8	1.3	1.3	1.1	1.2	1.9	1.3
Alteromonadales	Gammaproteobacteria	Proteobacteria	*Marinobacter sp. (Marinobacter hydrocarbonoclasticus)*	0.3	0.4	3.9	3.1	0.5	0.5	1.4	1.1	1.4	0.4	1.4	0.9	1.3	1.0	1.3	3.5	1.6
Enterobacteriales	Gammaproteobacteria	Proteobacteria	*Rahnella sp. (Rahnella aquatilis)*	0.8	0.6	1.8	1.6	1.5	0.8	0.5	0.3	1.2	1.0	1.2	1.3	1.1	1.8	1.5	1.3	1.4
Enterobacteriales	Gammaproteobacteria	Proteobacteria	*Serratia sp. (Serratia marcescens)*	0.6	0.6	1.8	1.6	0.9	0.9	1.1	0.9	1.0	0.6	0.8	0.9	1.1	1.0	1.2	1.7	1.1
Enterobacteriales	Gammaproteobacteria	Proteobacteria	*Yersinia sp. (Yersinia pseudotuberculosis)*	0.4	0.3	0.2	0.1	0.6	0.6	0.1	0.2	2.7	1.5	0.7	1.2	1.8	0.9	0.7	1.8	1.2
Rhizobiales	Alphaproteobacteria	Proteobacteria	*Methylobacterium sp. (Methylobacterium nodulans)*	0.8	1.2	0.7	0.8	1.0	0.9	1.5	1.3	0.8	0.8	1.7	0.6	0.9	1.0	1.2	1.0	1.1
Rhizobiales	Alphaproteobacteria	Proteobacteria	*Rhodopseudomonas sp. (Rhodopseudomonas palustris)*	1.2	1.2	0.9	1.0	0.8	0.8	1.3	1.3	1.0	0.8	2.4	1.0	1.0	1.0	1.0	1.2	1.3
Rhizobiales	Alphaproteobacteria	Proteobacteria	*Sinorhizobium sp. (Sinorhizobium medicae)*	0.5	0.6	0.8	0.7	1.2	1.2	1.5	1.4	0.9	1.1	2.2	0.8	1.1	1.0	1.0	0.8	1.2
Rhodobacterales	Alphaproteobacteria	Proteobacteria	*Roseobacter sp. (Roseobacter denitrificans)*	0.7	1.0	0.4	0.6	1.4	1.3	1.3	1.2	0.9	1.5	2.8	0.7	0.8	1.0	1.1	0.6	1.2
Rhodobacterales	Alphaproteobacteria	Proteobacteria	*Roseobacter sp. (Roseobacter litoralis)*	0.7	1.2	0.6	0.7	1.4	1.4	1.1	1.2	0.7	1.2	1.9	0.6	0.8	1.0	1.0	0.6	1.0
Rhodobacterales	Alphaproteobacteria	Proteobacteria	*Rhodobacter sp. (Rhodobacter sphaeroides)*	0.7	1.1	0.5	0.6	1.5	1.4	1.2	1.1	0.8	1.2	3.6	0.6	0.8	1.1	1.1	0.7	1.3
Rhodobacterales	Alphaproteobacteria	Proteobacteria	*Paracoccus sp. (Paracoccus denitrificans)*	0.7	1.1	0.6	0.7	1.3	1.3	1.2	1.2	0.8	1.1	3.8	0.6	0.8	1.0	1.0	0.7	1.3
Rhodobacterales	Alphaproteobacteria	Proteobacteria	*Jannaschia sp. (Jannaschia sp. CCS1)*	0.6	1.0	0.6	0.7	1.2	1.1	1.1	1.1	0.9	1.4	2.7	0.6	0.8	1.1	1.0	0.6	1.1
Rhodobacterales	Alphaproteobacteria	Proteobacteria	*Rhodobacter sp. (Rhodobacter capsulatus)*	0.7	1.1	0.4	0.6	1.3	1.2	1.2	1.1	0.9	1.2	3.2	0.7	0.8	1.1	1.1	0.7	1.2
Rhodobacterales	Alphaproteobacteria	Proteobacteria	*Ketogulonicigenium sp. (Ketogulonicigenium vulgare)*	0.7	1.0	0.5	0.7	1.2	1.1	1.1	1.1	0.7	1.0	2.3	0.7	0.8	1.1	1.0	0.7	1.1
Rhodobacterales	Alphaproteobacteria	Proteobacteria	*Paracoccus sp. (Paracoccus aminophilus)*	0.7	1.1	0.5	0.6	1.2	1.1	1.0	1.0	0.7	1.2	2.4	0.6	0.8	1.0	0.9	0.6	1.1
Rhodobacterales	Alphaproteobacteria	Proteobacteria	*Octadecabacter sp. (Octadecabacter antarcticus)*	0.7	1.2	0.4	0.6	0.9	0.9	0.9	1.0	0.7	1.3	1.4	0.6	0.8	1.0	0.9	0.6	0.9
Rhodobacterales	Alphaproteobacteria	Proteobacteria	*Octadecabacter sp. (Octadecabacter arcticus)*	0.7	1.0	0.5	0.7	0.9	1.0	0.9	1.0	0.7	1.3	1.4	0.7	0.8	0.9	0.9	0.5	0.9
Enterobacteriales	Gammaproteobacteria	Proteobacteria	*Erwinia sp. (Erwinia amylovora)*	1.1	1.1	1.4	1.4	0.5	0.4	1.7	1.0	1.0	0.5	1.3	1.0	1.0	1.3	1.6	2.1	1.4
Chlamydiales	Chlamydiia	Chlamydiae	*Simkania sp. (Simkania negevensis)*	1.7	1.1	0.2	0.1	0.3	0.3	1.8	1.7	1.3	1.3	8.6	1.5	1.4	0.9	1.0	1.0	2.4
Fusobacteriales	Fusobacteriia	Fusobacteria	*Fusobacterium sp. (Fusobacterium nucleatum)*	1.7	0.7	2.0	2.2	0.3	0.1	1.5	0.3	1.3	0.4	0.1	2.3	0.9	2.3	5.7	2.9	2.4
Desulfobacterales	Deltaproteobacteria	Proteobacteria	*Desulfobacula sp. (Desulfobacula toluolica)*	0.4	0.3	0.2	0.2	1.6	1.2	2.3	2.3	0.7	0.9	1.6	1.0	0.8	1.3	1.0	0.8	1.1
Campylobacterales	Epsilonproteobacteria	Proteobacteria	*Sulfurimonas sp. (Sulfurimonas denitrificans)*	0.1	0.1	2.0	1.0	0.2	0.2	1.9	4.1	0.9	1.1	0.8	1.5	2.0	1.0	0.5	0.8	1.1
Rhodospirillales	Alphaproteobacteria	Proteobacteria	*Azospirillum sp. (Azospirillum lipoferum)*	0.5	0.5	0.5	0.5	0.8	1.0	1.2	1.0	0.8	0.7	2.7	1.0	1.0	0.8	1.2	1.0	1.3
Rhodospirillales	Alphaproteobacteria	Proteobacteria	*Magnetospirillum sp. (Magnetospirillum gryphiswaldense)*	0.3	0.3	0.5	0.4	0.5	0.6	1.7	1.3	0.8	0.6	3.7	1.1	1.2	0.8	1.3	1.5	1.6
Sphingomonadales	Alphaproteobacteria	Proteobacteria	*Sphingomonas sp. (Sphingomonas wittichii)*	1.4	1.7	0.7	0.9	0.5	0.7	0.6	0.7	0.7	0.9	2.6	0.8	0.8	0.7	0.8	0.7	1.1
Sphingomonadales	Alphaproteobacteria	Proteobacteria	*Sphingopyxis sp. (Sphingopyxis alaskensis)*	1.5	2.0	0.6	0.7	0.3	0.4	0.6	0.8	0.7	1.0	2.0	0.7	0.8	0.7	0.8	0.6	1.0
Sphingomonadales	Alphaproteobacteria	Proteobacteria	*Sphingomonas sp. (Sphingomonas sp. MM-1)*	1.5	2.0	0.7	0.8	0.3	0.4	0.6	0.6	0.6	0.7	1.9	0.7	0.9	0.8	0.9	0.8	1.0
Sphingomonadales	Alphaproteobacteria	Proteobacteria	*Novosphingobium sp. (Novosphingobium sp. PP1Y)*	1.1	1.6	0.6	0.7	0.3	0.4	0.6	0.7	0.5	0.8	1.9	0.7	0.8	0.8	0.8	0.7	0.9

Species abundance

Supplementary Fig. 8 Bacterial aerosolization of abundance trimmed data set. (continued).

c

Enriched ← Neutral Transfer → Diminished

> 5.0	1.5 - 5.0	1.0 - 1.5	0.67 - 1.0	0.2 - 0.67	< 0.2

Order	Class	Phylum	Species	Aerosolization 8A:B	8A:S	19A:B	19A:S	24A:B	24A:S	28A:B	28A:S	34A:B	34A:S	Surface Enhancement 0S:B	8S:B	19S:B	24S:B	28S:B	34S:B	AVG
Sphingomonadales	Alphaproteobacteria	Proteobacteria	*Zymomonas sp. (Zymomonas mobilis)*	0.6	0.7	0.6	0.6	0.2	0.3	0.6	0.6	0.6	0.8	0.8	0.9	1.0	0.8	1.0	0.8	0.9
Sphingomonadales	Alphaproteobacteria	Proteobacteria	*Erythrobacter sp. (Erythrobacter litoralis)*	0.6	0.8	0.4	0.5	0.2	0.2	0.5	0.6	0.4	0.7	1.8	0.7	0.8	0.7	0.8	0.6	0.9
Cytophagales	Cytophagia	Bacteroidetes	*Cytophaga sp. (Cytophaga hutchinsonii)*	0.4	0.2	0.5	0.4	0.4	0.6	0.5	1.0	4.5	2.8	0.3	2.1	1.1	0.7	0.5	1.6	1.0
Cytophagales	Cytophagia	Bacteroidetes	*Cyclobacterium sp. (Cyclobacterium marinum)*	0.2	0.3	0.1	0.1	0.6	0.8	2.1	2.2	0.4	0.5	2.4	0.7	1.0	0.8	0.9	0.7	1.1
Flavobacteriales	Flavobacteriia	Bacteroidetes	*Capnocytophaga sp. (Capnocytophaga ochracea)*	0.2	0.3	0.4	0.4	0.1	0.1	1.2	1.7	0.6	1.8	1.5	0.8	0.9	0.7	0.7	0.4	0.8
Natrialbales	Halobacteria	Euryarchaeota	*Halovivax sp. (Halovivax ruber)*	1.1	0.7	1.6	0.6	0.4	0.2	0.4	0.2	1.9	0.5	0.4	1.7	2.7	2.3	2.4	3.8	2.2
Flavobacteriales	Flavobacteriia	Bacteroidetes	*Zobellia sp. (Zobellia galactanivorans)*	0.2	0.3	0.1	0.1	0.5	0.4	1.3	2.0	0.6	1.0	2.5	0.9	1.0	1.2	0.7	0.6	1.1
Flavobacteriales	Flavobacteriia	Bacteroidetes	*Blattabacterium sp. (Mastotermes darwiniensis)*	1.0	2.3	0.4	0.5	0.4	0.4	0.2	0.4	0.1	0.4	0.8	0.4	0.9	0.9	0.7	0.3	0.7
Flavobacteriales	Flavobacteriia	Bacteroidetes	*Ornithobacterium sp. (Ornithobacterium rhinotracheale)*	0.3	0.4	0.1	0.1	0.4	0.4	1.4	1.6	0.3	1.1	1.8	0.9	1.1	1.1	0.9	0.3	1.0
Flavobacteriales	Flavobacteriia	Bacteroidetes	*Polaribacter sp. (Polaribacter sp. MED152)*	0.3	0.3	0.1	0.1	0.2	0.2	0.8	1.3	0.2	2.3	3.1	0.9	1.0	1.0	0.6	0.1	1.1
Flavobacteriales	Flavobacteriia	Bacteroidetes	*Lacinutrix sp. (Lacinutrix sp. 5H-3-7-4)*	0.3	0.3	0.2	0.2	0.4	0.3	1.0	1.6	0.3	1.1	2.4	0.7	0.9	1.0	0.7	0.2	1.0
Flavobacteriales	Flavobacteriia	Bacteroidetes	*Nonlabens sp. (Nonlabens dokdonensis)*	0.1	0.2	0.3	0.3	0.4	0.3	1.0	1.5	0.2	0.7	1.9	0.8	1.0	1.1	0.7	0.4	1.0
Sphingobacteriales	Sphingobacteriia	Bacteroidetes	*Pedobacter sp. (Pedobacter saltans)*	0.4	0.2	0.2	0.2	0.2	0.2	1.5	2.3	0.3	0.8	2.6	1.7	1.0	0.8	0.6	0.4	1.2
Chlamydiales	Chlamydiia	Chlamydiae	*Waddlia sp. (Waddlia chondrophila)*	0.9	0.8	0.2	0.1	0.2	0.1	1.5	1.2	0.7	2.2	5.7	1.2	1.8	1.5	1.2	0.3	1.9
Thermales	Deinococci	Deinococcus-Thermus	*Meiothermus sp. (Meiothermus silvanus)*	0.3	0.3	0.6	0.6	0.2	0.2	1.6	0.8	0.2	0.1	3.2	1.1	1.0	1.2	1.9	2.3	1.8
Acidothermales	Actinobacteria	Actinobacteria	*Acidothermus sp. (Acidothermus cellulolyticus)*	0.4	0.3	0.9	0.8	0.5	0.4	0.3	0.4	0.3	0.3	0.8	1.5	1.2	1.1	0.8	1.1	1.1
Corynebacteriales	Actinobacteria	Actinobacteria	*Corynebacterium sp. (Corynebacterium kroppenstedtii)*	0.8	0.5	0.3	0.2	0.2	0.3	0.6	0.7	0.4	0.2	1.5	1.6	1.2	0.6	0.9	2.4	1.4
	Gammaproteobacteria	Proteobacteria	*Simiduia sp. (Simiduia agarivorans)*	0.3	0.3	1.2	0.9	0.5	0.5	1.0	0.8	1.2	0.8	1.8	1.3	1.4	1.0	1.3	1.4	1.4
	Epsilonproteobacteria	Proteobacteria	*Sulfurovum sp. (Sulfurovum sp. NBC37-1)*	0.5	0.3	0.1	0.1	0.1	0.1	0.9	1.6	0.2	0.7	1.3	1.4	1.0	0.6	0.5	0.4	0.9
Magnetococcales	Alphaproteobacteria	Proteobacteria	*Magnetococcus sp. (Magnetococcus marinus)*	0.2	0.1	0.5	0.5	0.4	0.4	0.8	0.7	0.6	0.6	2.6	1.2	1.1	0.9	1.3	1.0	1.4
	Alphaproteobacteria	Proteobacteria	*Polymorphum sp. (Polymorphum gilvum)*	0.6	0.7	0.6	0.7	1.2	1.2	1.4	1.4	0.8	0.8	3.3	0.8	0.9	0.9	1.1	0.9	1.3
	Alphaproteobacteria	Proteobacteria	*alpha sp. (alpha proteobacterium HIMB59)*	0.4	0.3	0.2	0.2	0.4	0.3	0.4	0.2	0.5	0.8	0.1	1.4	1.3	1.1	2.1	0.7	1.1
Nitrosomonadales	Betaproteobacteria	Proteobacteria	*Nitrosospira sp. (Nitrosospira multiformis)*	0.2	0.2	0.9	0.9	0.6	0.7	0.8	0.7	0.1	0.1	3.3	1.2	1.0	0.8	1.1	1.7	1.5
Desulfovibrionales	Deltaproteobacteria	Proteobacteria	*Desulfohalobium sp. (Desulfohalobium retbaense)*	0.6	0.4	0.6	0.7	0.5	0.5	0.6	0.6	0.5	0.5	1.8	1.6	0.9	1.0	1.1	1.0	1.2
Desulfuromonadales	Deltaproteobacteria	Proteobacteria	*Geobacter sp. (Geobacter sulfurreducens)*	0.2	0.2	1.1	1.1	0.5	0.5	0.9	0.7	0.9	0.5	2.3	1.0	0.9	1.0	1.4	1.8	1.4
Desulfuromonadales	Deltaproteobacteria	Proteobacteria	*Geobacter sp. (Geobacter lovleyi)*	0.7	1.2	0.6	0.6	0.2	0.2	0.6	0.3	0.3	0.3	1.1	0.6	1.0	0.8	2.1	0.8	1.1
Desulfuromonadales	Deltaproteobacteria	Proteobacteria	*Geobacter sp. (Geobacter daltonii)*	0.2	0.2	0.3	0.4	0.2	0.4	0.6	0.6	0.5	0.8	1.0	0.8	0.8	0.5	1.0	0.7	0.8
Campylobacterales	Epsilonproteobacteria	Proteobacteria	*Nitratifractor sp. (Nitratifractor salsuginis)*	0.2	0.2	0.9	0.9	0.6	0.5	0.2	0.2	0.4	0.7	0.5	1.3	1.0	1.2	1.0	0.7	0.9
Campylobacterales	Epsilonproteobacteria	Proteobacteria	*Arcobacter sp. (Arcobacter nitrofigilis)*	0.6	0.6	0.3	0.2	0.4	0.3	0.3	0.3	0.5	0.7	0.3	1.1	1.2	1.2	0.8	0.7	0.9
Alteromonadales	Gammaproteobacteria	Proteobacteria	*Shewanella sp. (Shewanella sp. MR-7)*	0.2	0.1	1.4	0.7	0.4	0.3	1.2	0.6	1.3	0.6	1.1	1.4	2.0	1.1	2.0	2.1	1.6
Alteromonadales	Gammaproteobacteria	Proteobacteria	*Psychromonas sp. (Psychromonas sp. CNPT3)*	0.6	0.6	0.7	0.4	0.5	0.4	0.6	0.5	0.6	0.4	0.5	0.9	1.7	1.3	1.1	1.4	1.2
Alteromonadales	Gammaproteobacteria	Proteobacteria	*Idiomarina sp. (Idiomarina loihiensis)*	0.6	0.6	0.6	0.4	0.3	0.2	0.8	0.5	0.7	0.5	1.0	1.1	1.6	1.7	1.4	1.3	1.4
Enterobacteriales	Gammaproteobacteria	Proteobacteria	*Edwardsiella sp. (Edwardsiella ictaluri)*	0.4	0.3	1.1	0.9	0.4	0.3	0.5	0.4	0.7	0.3	0.5	1.2	1.3	1.2	1.2	2.0	1.2
Legionellales	Gammaproteobacteria	Proteobacteria	*Coxiella sp. (Coxiella burnetii)*	0.6	0.6	1.0	0.6	0.2	0.4	0.6	0.8	0.4	0.4	1.1	1.0	1.6	0.5	0.8	1.0	1.0
Pasteurellales	Gammaproteobacteria	Proteobacteria	*Haemophilus sp. (Haemophilus parasuis)*	0.7	0.6	0.3	0.3	0.5	0.3	0.6	0.5	1.2	0.6	0.8	1.0	1.1	1.7	1.3	1.8	1.3
Pseudomonadales	Gammaproteobacteria	Proteobacteria	*Moraxella sp. (Moraxella catarrhalis)*	0.4	0.3	0.2	0.1	0.4	0.3	1.0	0.7	0.5	0.3	0.6	1.3	1.3	1.3	1.5	1.4	1.2
Thiotrichales	Gammaproteobacteria	Proteobacteria	*Francisella sp. (Francisella sp. TX077308)*	0.5	0.6	0.6	0.5	0.5	0.5	0.7	0.9	0.4	0.2	0.7	0.8	1.4	1.0	0.8	2.3	1.2
Burkholderiales	Betaproteobacteria	Proteobacteria	*Ralstonia sp. (Ralstonia pickettii)*	0.9	0.9	1.5	1.4	0.8	0.9	0.9	0.7	1.1	0.9	1.7	1.0	1.1	0.9	1.3	1.2	1.2
Burkholderiales	Betaproteobacteria	Proteobacteria	*Taylorella sp. (Taylorella asinigenitalis)*	0.1	0.1	1.1	1.0	0.1	0.1	0.5	0.5	0.7	1.2	0.8	1.1	1.2	0.9	1.2	0.6	1.0
Burkholderiales	Betaproteobacteria	Proteobacteria	*Taylorella sp. (Taylorella equigenitalis)*	0.3	0.2	0.2	0.1	0.3	0.6	0.8	0.6	0.1	0.2	0.8	1.7	1.3	0.4	1.3	0.8	1.1
Bacteroidales	Bacteroidia	Bacteroidetes	*Bacteroides sp. (Bacteroides vulgatus)*	0.6	0.8	0.8	0.7	0.5	0.7	0.6	0.8	0.3	0.1	1.5	0.7	1.1	0.8	0.7	2.2	1.2
Bacteroidales	Bacteroidia	Bacteroidetes	*Candidatus Azobacteroides sp. (Candidatus Azobacteroides pseudotrichonymphae)*	0.8	0.3	0.6	0.5	0.4	0.4	0.4	0.5	0.7	0.6	1.0	2.5	1.1	0.8	0.7	1.1	1.2
Bacteroidales	Bacteroidia	Bacteroidetes	*Bacteroides sp. (Bacteroides fragilis)*	0.4	0.3	0.6	0.5	0.6	0.4	0.7	0.4	0.5	0.9	1.4	1.5	1.2	1.6	1.6	0.6	1.3
Bacteroidales	Bacteroidia	Bacteroidetes	*Paludibacter sp. (Paludibacter propionicigenes)*	0.4	0.7	0.1	0.1	0.5	0.6	0.7	1.0	0.2	0.3	0.9	0.7	0.9	0.9	0.7	0.8	0.8
Flavobacteriales	Flavobacteriia	Bacteroidetes	*Croceibacter sp. (Croceibacter atlanticus)*	0.3	0.4	0.2	0.2	0.4	0.4	0.7	1.2	0.3	1.5	1.6	0.7	1.0	0.9	0.6	0.2	0.8
Flavobacteriales	Flavobacteriia	Bacteroidetes	*Fluviicola sp. (Fluviicola taffensis)*	0.4	0.4	0.5	0.5	0.3	0.4	1.0	1.2	0.2	0.5	1.3	1.0	1.0	0.9	0.8	0.4	0.9
Flavobacteriales	Flavobacteriia	Bacteroidetes	*Blattabacterium sp. (Blattabacterium punctulatus)*	0.5	0.3	0.5	0.6	0.1	0.3	0.9	1.5	0.2	0.3	1.2	1.5	0.9	0.6	0.6	0.9	1.0
Flavobacteriales	Flavobacteriia	Bacteroidetes	*Dokdonia sp. (Dokdonia sp. 4H-3-7-5)*	0.3	0.3	0.1	0.2	0.4	0.4	0.8	1.0	0.3	1.1	2.1	0.8	0.9	1.1	0.7	0.3	1.0
Flavobacteriales	Flavobacteriia	Bacteroidetes	*Maribacter sp. (Maribacter sp. HTCC2170)*	0.2	0.2	0.1	0.1	0.4	0.3	0.9	1.3	0.3	0.9	2.3	0.8	0.9	1.2	0.6	0.3	1.0
Flavobacteriales	Flavobacteriia	Bacteroidetes	*Cellulophaga sp. (Cellulophaga algicola)*	0.2	0.3	0.2	0.2	0.3	0.3	0.8	1.1	0.3	1.2	1.8	0.9	1.1	1.0	0.7	0.2	1.0
Flavobacteriales	Flavobacteriia	Bacteroidetes	*Flavobacterium sp. (Flavobacterium indicum)*	0.2	0.3	0.2	0.2	0.2	0.2	0.9	1.2	0.2	1.2	2.3	0.7	1.0	0.9	0.7	0.2	1.0
Flavobacteriales	Flavobacteriia	Bacteroidetes	*Blattabacterium sp. (Blattella germanica)*	0.1	0.3	0.2	0.2	0.5	0.5	0.6	1.0	0.5	0.9	2.8	0.5	0.9	0.9	0.6	0.6	1.0
Flavobacteriales	Flavobacteriia	Bacteroidetes	*Owenweeksia sp. (Owenweeksia hongkongensis)*	0.2	0.2	0.2	0.2	0.3	0.3	0.7	1.0	0.4	1.3	1.8	0.8	0.9	0.9	0.7	0.3	0.9
Flavobacteriales	Flavobacteriia	Bacteroidetes	*Aequorivita sp. (Aequorivita sublithincola)*	0.1	0.2	0.3	0.3	0.4	0.3	0.8	1.2	0.4	0.8	1.5	0.8	1.0	1.1	0.7	0.5	0.9
Flavobacteriales	Flavobacteriia	Bacteroidetes	*Zunongwangia sp. (Zunongwangia profunda)*	0.2	0.3	0.3	0.3	0.3	0.3	0.9	1.1	0.3	0.6	2.5	0.7	0.9	1.0	0.8	0.4	1.0
Flavobacteriales	Flavobacteriia	Bacteroidetes	*Capnocytophaga sp. (Capnocytophaga canimorsus)*	0.1	0.2	0.2	0.2	0.4	0.3	1.0	1.4	0.3	0.7	2.1	0.7	0.9	1.2	0.7	0.5	1.0
Flavobacteriales	Flavobacteriia	Bacteroidetes	*Cellulophaga sp. (Cellulophaga lytica)*	0.3	0.4	0.2	0.2	0.3	0.3	0.8	1.1	0.2	0.8	1.9	0.8	1.0	0.9	0.7	0.2	0.9
Sphingobacteriales	Sphingobacteriia	Bacteroidetes	*Sphingobacterium sp. (Sphingobacterium sp. 21)*	0.3	0.6	0.3	0.4	0.4	0.3	0.8	1.3	0.1	0.1	1.3	0.5	0.9	1.3	0.6	0.6	0.9
Chlorobiales	Chlorobia	Chlorobi	*Prosthecochloris sp. (Prosthecochloris aestuarii)*	0.2	0.1	0.8	0.6	0.6	0.6	0.6	0.9	0.7	0.6	0.8	1.6	1.4	1.0	0.7	1.1	1.1
Herpetosiphonales	Chloroflexia	Chloroflexi	*Herpetosiphon sp. (Herpetosiphon aurantiacus)*	0.3	0.2	0.3	0.6	0.2	0.4	0.9	0.8	0.4	0.1	1.5	1.7	0.6	0.5	1.2	4.6	1.7
Oscillatoriales	Geitlerinema	Cyanobacteria	*Geitlerinema sp. (Geitlerinema sp. PCC 7407)*	0.3	0.3	0.8	0.5	0.6	0.7	0.6	0.3	0.3	0.1	0.9	1.2	1.8	0.9	1.7	2.0	1.4
Deinococcales	Deinococci	Deinococcus-Thermus	*Deinococcus sp. (Deinococcus deserti)*	0.4	0.3	0.3	0.2	0.7	0.6	0.5	0.4	0.2	0.4	1.8	1.2	1.1	1.2	1.2	0.6	1.2
Thermales	Deinococci	Deinococcus-Thermus	*Meiothermus sp. (Meiothermus ruber)*	0.6	0.4	0.5	0.5	0.2	0.2	0.9	0.7	0.5	0.6	1.0	1.5	1.2	0.9	1.4	0.7	1.1
Bacillales	Bacilli	Firmicutes	*Exiguobacterium sp. (Exiguobacterium sp. AT1b)*	0.2	0.3	0.3	0.2	0.4	0.2	0.3	0.4	0.6	0.6	1.2	0.6	1.3	1.7	0.8	1.1	1.1
Lactobacillales	Bacilli	Firmicutes	*Streptococcus sp. (Streptococcus equi)*	0.3	0.2	0.4	0.5	0.8	0.9	0.3	0.2	0.8	0.4	1.8	1.6	0.7	0.9	1.7	2.2	1.5
Clostridiales	Clostridia	Firmicutes	*Desulfotomaculum sp. (Desulfotomaculum kuznetsovii)*	0.6	0.4	0.6	0.3	0.2	0.4	0.9	0.8	0.3	0.1	0.8	1.6	1.8	0.6	1.2	2.3	1.4

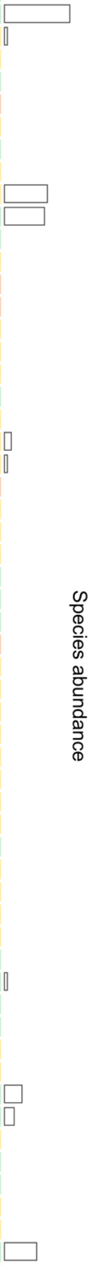

Supplementary Fig. 8 Bacterial aerosolization of abundance trimmed data set. (continued).

			Day	8		19		24		28		34			0	8	19	24	28	34		
Order	Family	Genus	Species / AF	A:B	A:S	A:B	A:S	A:B	A:S	A:B	A:S	A:B	A:S	Avg AF	S:B	S:B	S:B	S:B	S:B	S:B	Avg S:B	Abundance
Caudovirales	*Myoviridae*	*T4likevirus*	*Klebsiella* phage KP15	0.46	0.72	41.3	5.51	2.14	4.70	2.24	0.60	238	4.97	30.1	0.02	0.63	7.49	0.46	3.71	47.9	10.0	
Caudovirales	*Myoviridae*		*Klebsiella* phage KP27	0.61	0.62	6.60	4.41	1.70	5.20	2.44	0.62	146	8.86	17.7	0.05	0.98	1.50	0.33	3.95	16.4	3.87	
Caudovirales	*Myoviridae*	*T4likevirus*	*Prochlorococcus* phage P-SSM2	0.08	0.02	0.94	0.37	0.08	0.08	4.07	0.45	7.88	0.73	1.47	0.07	5.00	2.54	1.05	9.06	10.8	4.75	
Caudovirales	*Myoviridae*	*T4likevirus*	*Synechococcus* phage metaG-MbCM1	0.09	0.03	0.15	0.15	0.06	0.08	6.11	0.23	19.5	1.43	2.79	0.32	3.36	1.04	0.71	26.4	13.7	7.58	
Caudovirales	*Myoviridae*	*T4likevirus*	*Synechococcus* phage S-MbCM100	0.11	0.02	0.89	0.55	0.19	0.34	1.36	0.69	10.3	0.73	1.52	0.43	4.62	1.64	0.56	1.98	14.1	3.89	
Caudovirales	*Myoviridae*		*Pelagibacter* phage HTVC008M	0.11	0.03	0.30	0.24	0.17	0.10	3.03	0.26	6.70	0.34	1.13	0.35	3.49	1.23	1.67	11.7	19.8	6.37	
Caudovirales	*Myoviridae*		*Prochlorococcus* phage MED4-213	0.26	0.02	1.65	1.18	0.09	0.43	2.04	0.62	6.76	0.45	1.35	0.46	11.0	1.40	0.21	3.30	15.2	5.26	
Caudovirales	*Myoviridae*		*Prochlorococcus* phage P-SSM7	0.17	0.04	0.59	0.39	0.25	0.40	0.00	0.00	7.33	0.48	0.96	0.31	4.27	1.50	0.62	6.59	15.4	4.78	
Caudovirales	*Myoviridae*		*Synechococcus* phage S-ShM2	0.15	0.02	0.90	0.81	0.11	0.08	3.06	1.85	10.8	0.44	1.82	0.24	7.86	1.11	1.44	1.65	24.7	6.16	
Caudovirales	*Myoviridae*		*Synechococcus* phage S-SM2	0.10	0.03	0.26	0.18	0.14	0.09	1.08	0.15	9.66	0.63	1.23	0.25	4.08	1.47	1.63	7.07	15.3	4.97	
Caudovirales	*Myoviridae*		*Synechococcus* phage S-SSM7	0.02	0.00	1.10	0.37	0.07	0.13			12.5	0.67	1.86	0.19	4.59	2.96	0.52	44.5	18.6	11.9	
Caudovirales	*Podoviridae*	*Phikmvlikevirus*	*Pseudomonas* phage LKA1	1.13	1.68	19.5	6.42	3.62	8.22	1.90	0.63	196	7.05	24.6	0.09	0.68	3.04	0.44	3.01	27.8	5.84	
Caudovirales	*Podoviridae*		*Pelagibacter* phage HTVC010P	0.08	0.03	0.21	0.21	0.13	0.06	2.65	0.19	4.42	0.36	0.83	0.70	2.41	0.98	2.03	13.7	12.3	5.35	
Caudovirales	*Podoviridae*		*Pelagibacter* phage HTVC019P	0.08	0.03	0.17	0.20	0.16	0.12	2.72	0.17	7.36	0.87	1.19	0.39	2.92	0.87	1.30	15.7	8.43	4.93	
Caudovirales	*Podoviridae*		*Puniceispirillum* phage HMO-2011	0.07	0.02	0.19	0.22	0.10	0.04	2.05	0.17	6.32	0.33	0.95	0.61	3.49	0.89	2.25	12.4	19.1	6.46	
Caudovirales	*Podoviridae*		*Synechococcus* phage S-RIP1	0.07	0.02	0.11	0.14	0.15	0.14	3.06	0.37	9.06	0.51	1.36	0.97	3.02	0.74	1.06	8.24	17.9	5.33	
Caudovirales	*Siphoviridae*		*Synechococcus* phage S-SKS1	0.10	0.03	0.42	0.25	0.10	0.10	3.91	0.36	10.3	0.63	1.62	0.15	3.15	1.69	0.99	11.0	16.4	5.57	
Caudovirales			*Prochlorococcus* phage Syn1	0.09	0.03	0.52	0.29	0.05	0.05			22.4	2.14	3.19	0.28	3.50	1.80	1.10	7.69	10.5	4.14	
Caudovirales			*Prochlorococcus* phage Syn33	0.11	0.03	0.26	0.20	0.06	0.14	1.63	0.25	12.4	1.43	1.65	0.30	3.28	1.29	0.44	6.59	8.70	3.43	
	Inovirdae	*Inovirus*	Enterobacteria phage M13	0.66	2.87	0.15	0.22	3.76	1.54	0.04	0.46	0.11	0.13	0.99	0.22	0.23	0.68	2.43	0.08	0.85	0.75	
			Prochlorococcus phage P-GSP1	0.05	0.01	0.50	0.46	0.06	0.05	6.11	0.21	5.34	0.28	1.31	0.11	3.72	1.11	1.17	29.7	19.4	9.19	
	Phycodnaviridae	*Prasinovirus*	*Ostreococcus lucimarinus virus* OlV1	0.06	0.03	0.26	0.33	0.08	0.12	0.58	0.27	5.55	0.80	0.81	0.64	2.40	0.80	0.64	2.12	6.92	2.25	
	Phycodnaviridae	*Prymnesiovirus*	*Phaeocystis globosa virus*	5.42	6.65	0.29	0.53	1.58	0.59	1.45	1.06	6.17	1.10	2.48	1.97	0.82	0.55	2.69	1.37	5.62	2.17	
	Polydnaviridae	*Bracovirus*	*Cotesia congregata bracovirus*	5.40	4.96	0.35	0.73	1.81	0.64	1.71	0.88	8.66	1.73	2.69	1.22	1.09	0.48	2.82	1.94	5.00	2.09	
	Polydnaviridae	*Ichnovirus*	*Glypta fumiferanae ichnovirus*	5.26	7.24	0.41	0.48	2.27	0.66	1.85	1.23	4.75	0.99	2.51	2.23	0.73	0.85	3.43	1.51	4.78	2.26	
	Poxviridae	*Molluscipoxvirus*	*Molluscum contagiosum virus*	7.33		0.83	0.81	2.31	0.46	0.94	0.41		1.25	1.79	1.59	0.27	1.01	5.07	2.28		2.04	
Herpesvirales	*Alloherpesviridae*	*Cyprinivirus*	*Cyprinid herpesvirus* 1	1.77	32.6	1.20	0.59	1.41	0.23	1.73	0.64	1.74	1.43	4.33	0.57	0.05	2.04	6.19	2.72	1.22	2.13	
Herpesvirales	*Alloherpesviridae*	*Cyprinivirus*	*Cyprinid herpesvirus* 3	2.71	6.33	0.79	0.84	1.15	0.46	1.54	0.68	2.47	1.14	1.81	0.70	0.43	0.94	2.54	2.27	2.16	1.51	
Herpesvirales	*Alloherpesviridae*	*Ictalurivirus*	*Ictalurid herpesvirus* 1	5.99	4.40	0.66	1.41	2.24	0.88	1.59	1.31	6.87	0.81	2.62	2.95	1.36	0.47	2.56	1.21	8.50	2.84	
Herpesvirales	*Herpesviridae*	*Roseolovirus*	*Human herpesvirus* 6A		8.24	0.83	0.13		1.03	4.07	1.16	2.34	1.07	2.36	1.05		6.32		3.50	2.19	3.27	

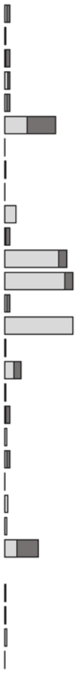

Aerosolization (A:B, A:S columns, days 8–34) — Surface Enhancement (S:B columns, days 0–34)

Legend: >5.0 | 1.5 - 5.0 | 1.0 - 1.5 | 0.67 - 1.0 | 0.2 - 0.67 | <0.2

Enriched ← Neutral Transfer → Diminished

Total fraction | Samples > 0.5%

Supplementary Fig. 9 Viral aerosolization and surface enhancement. Taxonomy, aerosolization factors, and surface enrichment of viral genomes in the abundance and genome coverage trimmed data set. Heat map column labels indicate day and measurement. Aerosolization factor is expressed as the fraction of genomes in aerosol to bulk or surface (A:B or A:S). Surface enrichment is the fraction in SSML to the fraction in the bulk (S:B). Heat map values indicate ratio values (bottom legend). Genome abundance is reported as the sum of fraction of the population across the experiment and as the number of samples above 0.5% of the population. Blanks indicate samples below threshold limit.

2. Supplementary Tables

Supplementary Table 1 DNA extraction yields

Sample Date	Sample Type	Fraction	Total DNA recovered (ng)	Sample DNA concentration (ng mL^{-1})*	Sample Date	Sample Type	Fraction	Total DNA recovered (ng)	Sample DNA concentration (ng/mL^{-1})*
7/3/14	Bulk	3µm	1914.00	1.91	7/27/14	Bulk	3µm	1152.00	5.76E-01
7/3/14	Bulk	0.2µm	2850.00	1.43	7/27/14	Bulk	0.2µm	636.00	3.18E-01
7/3/14	Bulk	0.025µm	73.44	1.47E-01	7/27/14	Bulk	0.025µm	335.20	3.35E-01
7/3/14	SSML	3µm	307.20	1.54	7/27/14	SSML	3µm	765.60	3.83
7/3/14	SSML	0.2µm	628.80	3.14	7/27/14	SSML	0.2µm	1195.20	5.98
7/3/14	SSML	0.025µm	2.60	1.30E-02	7/27/14	SSML	0.025µm	251.33	1.26
7/5/14	Aerosol	3µm	0.75	5.22E-07	7/27/14	Aerosol	3µm	3.20	1.44E-06
7/5/14	Aerosol	0.2µm	0.80	5.53E-07	7/27/14	Aerosol	0.2µm	21.76	9.07E-06
7/5/14	Aerosol	0.025µm	8.01	3.66E-06	7/27/14	Aerosol	0.025µm	2.72	1.10E-06
7/11/14	Bulk	3µm	554.40	2.77E-01	7/31/14	Bulk	3µm	991.20	4.96E-01
7/11/14	Bulk	0.2µm	567.60	2.84E-01	7/31/14	Bulk	0.2µm	1248.00	8.91E-01
7/11/14	Bulk	0.025µm	4.61	4.61E-03	7/31/14	Bulk	0.025µm	344.40	3.44E-01
7/11/14	SSML	3µm	244.80	1.22	7/31/14	SSML	3µm	456.00	2.28
7/11/14	SSML	0.2µm	342.40	1.71	7/31/14	SSML	0.2µm	624.00	3.12
7/11/14	SSML	0.025µm	71.34	3.57E-01	7/31/14	SSML	0.025µm	305.87	1.53
7/11/14	Aerosol	3µm	6.09	2.78E-06	7/31/14	Aerosol	3µm	0.91	3.80E-07
7/11/14	Aerosol	0.2µm	220.73	7.93E-05	7/31/14	Aerosol	0.2µm	2.81	1.17E-06
7/11/14	Aerosol	0.025µm	3.38	1.45E-06	7/31/14	Aerosol	0.025µm	1.41	5.87E-07
7/22/14	Bulk	3µm	559.20	2.80E-01	8/6/14	Bulk	3µm	583.20	2.92E-01
7/22/14	Bulk	0.2µm	1344.00	6.72E-01	8/6/14	Bulk	0.2µm	696.00	3.48E-01
7/22/14	Bulk	0.025µm	375.20	3.75E-01	8/6/14	Bulk	0.025µm	584.40	5.84E-01
7/22/14	SSML	3µm	274.40	1.37	8/6/14	SSML	3µm	122.64	6.13E-01
7/22/14	SSML	0.2µm	342.40	1.71	8/6/14	SSML	0.2µm	378.00	1.89
7/22/14	SSML	0.025µm	248.00	1.24	8/6/14	SSML	0.025µm	115.44	5.77E-01
7/22/14	Aerosol	3µm	3.02	1.26E-06	8/6/14	Aerosol	3µm	1.26	5.23E-07
7/22/14	Aerosol	0.2µm	5.53	2.30E-06	8/6/14	Aerosol	0.2µm	3.06	1.28E-06
7/22/14	Aerosol	0.025µm	2.59	1.20E-06	8/6/14	Aerosol	0.025µm	1.06	1.15E-06

*Bulk and SSML measurements are per mL of seawater, aerosol is per mL of air

Supplementary Table 2 Trimmed and filtered read statistics

Sample Name	Sequences (M)	Length (bp)	Percent GC	Sample Name	Sequences (M)	Length (bp)	Percent GC
03B0025_R1	1.7	138	46%	27B0025_R1	1.9	138	43%
03B0025_R2	1.7	147	46%	27B0025_R2	1.9	145	43%
03B02_R1	4.1	147	46%	27B3_R1	5.4	136	48%
03B02_R2	4.1	137	46%	27B3_R2	5.4	147	48%
03B3_R1	4.8	147	47%	27S0025_R1	4.2	137	44%
03B3_R2	4.8	138	47%	27S0025_R2	4.2	148	44%
03S0025_R1	1.3	136	46%	27S02_R1	5.4	148	51%
03S0025_R2	1.3	147	46%	27S02_R2	5.4	138	51%
03S02_R1	1.5	138	46%	27S3_R1	5.0	137	46%
03S02_R2	1.5	147	46%	27S3_R2	5.0	147	46%
03S3_R1	1.1	147	46%	31A0025_R1	1.1	147	48%
03S3_R2	1.1	138	46%	31A0025_R2	1.1	136	48%
05A0025_R1	0.8	137	48%	31A02_R1	1.3	147	48%
05A0025_R2	0.8	147	48%	31A02_R2	1.3	137	48%
05A02_R1	2.3	138	48%	31A3_R1	0.7	137	47%
05A02_R2	2.3	147	48%	31A3_R2	0.7	146	47%
05A3_R1	1.7	147	45%	31B0025_R1	1.4	147	47%
05A3_R2	1.7	137	45%	31B0025_R2	1.4	137	47%
11A0025_R1	1.0	147	46%	31B02_R1	6.4	148	49%
11A0025_R2	1.0	137	46%	31B02_R2	6.4	138	49%
11A02_R1	6.8	148	52%	31B3_R1	8.7	137	48%
11A02_R2	6.8	139	52%	31B3_R2	8.7	147	48%
11A3_R1	3.3	147	52%	31S0025_R1	2.3	136	45%
11A3_R2	3.3	138	52%	31S0025_R2	2.3	147	45%
11B0025_R1	0.7	138	44%	31S02_R1	10.7	148	51%
11B0025_R2	0.7	147	44%	31S02_R2	10.7	139	51%
11B02_R1	5.3	148	51%	31S3_R1	1.7	146	48%
11B02_R2	5.3	138	51%	31S3_R2	1.7	134	48%
11B3_R1	2.0	147	50%	86_27B02_R1	9.7	139	51%
11B3_R2	2.0	138	50%	86_27B02_R2	9.7	148	51%
11S0025_R1	5.7	148	43%	86A0025_R1	1.5	147	49%
11S0025_R2	5.7	140	43%	86A0025_R2	1.5	138	49%
11S02_R1	1.6	147	51%	86A02_R1	1.1	147	50%
11S02_R2	1.6	135	51%	86A02_R2	1.1	137	50%
11S3_R1	1.4	137	49%	86A3_R1	1.0	147	49%
11S3_R2	1.4	147	49%	86A3_R2	1.0	138	49%
18B3_02_R1	1.8	136	47%	86Ad_R1	1.4	147	48%
18B3_02_R2	1.8	147	47%	86Ad_R2	1.4	137	48%
22A0025_R1	0.7	147	45%	86B0025_R1	8.6	148	53%
22A0025_R2	0.7	137	45%	86B0025_R2	8.6	140	53%
22A02_R1	1.0	137	49%	86B3_R1	6.1	136	49%
22A02_R2	1.0	147	49%	86B3_R2	6.1	147	49%
22A3_R1	1.0	137	46%	86Bd_R1	1.1	138	50%
22A3_R2	1.0	147	46%	86Bd_R2	1.1	147	50%
22B0025_R1	2.5	137	43%	86S0025_R1	2.1	147	47%
22B0025_R2	2.5	148	43%	86S0025_R2	2.1	138	47%
22B02_R1	9.3	140	49%	86S02_R1	1.0	138	51%
22B02_R2	9.3	148	49%	86S02_R2	1.0	147	51%
22B3_R1	4.6	139	45%	86S3_R1	1.3	146	47%
22B3_R2	4.6	147	45%	86S3_R2	1.3	135	47%
22S0025_R1	4.7	148	43%	86Sd_R1	1.3	137	48%
22S0025_R2	4.7	138	42%	86Sd_R2	1.3	147	48%
22S02_R1	6.8	138	48%	87CA02_R1	0.6	137	46%
22S02_R2	6.8	148	49%	87CA02_R2	0.6	146	46%
22S3_R1	2.1	147	46%	87CA3_R1	4.7	147	51%
22S3_R2	2.1	137	46%	87CA3_R2	4.7	137	51%
27_86B02_R1	2.9	148	51%	88B02_R1	2.5	136	50%
27_86B02_R2	2.9	136	51%	88B02_R2	2.5	147	50%
27A0025_R1	1.0	137	47%	88B3_R1	1.0	138	47%
27A0025_R2	1.0	147	47%	88B3_R2	1.0	147	47%
27A02_R1	3.2	137	54%	88BS3_02_R1	1.6	147	48%
27A02_R2	3.2	147	54%	88BS3_02_R2	1.6	137	47%
27A3_R1	0.9	138	50%	mbla_R1	1.7	138	49%
27A3_R2	0.9	147	50%	mbla_R2	1.7	147	49%
AVERAGE	3.0	142.3	47.9%				
MIN	0.6	134.0	42.0%				
MAX	10.7	148.0	54.0%				

Supplementary Table 3 Spatial coverage of scaffolds to Kraken assigned genomes

Taxonomy	scaffold size (nts)	spatial coverage (nts)	spatial coverage (%)
Acinetobacter calcoaceticus	4110074	17174	0.4%
Alcanivorax dieselolei	4928223	203641	4.1%
alpha proteobacterium HIMB5	1343202	4965	0.4%
alpha proteobacterium HIMB59	1410127	21163	1.5%
Alteromonas macleodii	4575623	1917348	41.9%
Bacillus cereus	5295158	186859	3.5%
Bacteroides vulgatus	5163189	27842	0.5%
Blastococcus saxobsidens	4875340	9395	0.2%
Bradyrhizobium diazoefficiens	9105828	10991	0.1%
Burkholderia xenovorans	3243713	13728	0.4%
Candidatus Pelagibacter ubique	1395018	27281	2.0%
Candidatus Puniceispirillum marinum	2753527	33134	1.2%
Cellulophaga algicola	4888353	8416	0.2%
Cellulophaga lytica	3824196	20408	0.5%
Corynebacterium aurimucosum	1409613	23234	4.8%
Corynebacterium kroppenstedtii	2446804	4844	0.2%
Cotesia congregata bracovirus	28766	157.5	0.7%
Croceibacter atlanticus	2952962	14005	0.5%
Cyclobacterium marinum	6221273	15968	0.3%
Cyprinid herpesvirus 1	291144	1044	0.4%
Cyprinid herpesvirus 3	295146	316	0.1%
Dinoroseobacter shibae	869114.4	39816.4	25.4%
Enterobacteria phage M13	6407	636	9.9%
Erythrobacter litoralis	3251353	3890	0.1%
Flavobacterium indicum	2993089	5620	0.2%
Fusobacterium nucleatum	2443126	3274	0.1%
Geodermatophilus obscurus	5322497	18786	0.4%
Glypta fumiferanae ichnovirus	3141	568	18.1%
Gordonia polyisoprenivorans	2922149.5	1953	0.2%
Human herpesvirus 6A	156714	363	0.2%
Ictalurid herpesvirus 1	134226	321	0.2%
Idiomarina loihiensis	2839759	13167	0.5%
Ketogulonicigenium vulgare	1516261	4392	0.4%
Klebsiella phage KP15	174436	87598	50.2%
Klebsiella phage KP27	174413	73104	41.9%
Legionella pneumophila	3409143	55709	1.6%
Leisingera methylohalidivorans	1550332	5559.67	0.2%
Marinobacter adhaerens	2304688	488703.5	17.6%
Marinobacter hydrocarbonoclasticus	3989480	411335	10.3%
Methylobacterium radiotolerans	1685848	319342.25	10.3%
Methylophaga frappieri	1372645	27145	3.5%
Methylophaga nitratireducenticrescens	3137191	656987	20.9%
Micrococcus luteus	2501097	302946	12.1%
Molluscum contagiosum virus	190289	709	0.4%
Moraxella catarrhalis	1887974	4629	0.2%
Mycobacterium avium	5011264	12848	0.3%
Mycobacterium chubuense	3099500.5	1291.5	0.2%
Nakamurella multipartita	6060298	245383	4.0%
Nonlabens dokdonensis	3914632	8829	0.2%
Novosphingobium aromaticivorans	1873023	17855	1.7%
Octadecabacter antarcticus	4812600	5381	0.1%
Octadecabacter arcticus	5200279	5393	0.1%
Ostreococcus lucimarinus virus OlV1	194022	69241	35.7%
Paracoccus denitrificans	2291189.5	9244.5	0.4%
Pelagibacter phage HTVC008M	147284	92310	62.7%
Pelagibacter phage HTVC010P	34892	23275	66.7%
Pelagibacter phage HTVC019P	42084	16404	39.0%
Phaeobacter gallaeciensis	4061725	13031	0.3%
Phaeobacter inhibens	2041825.5	7261.5	0.5%
Phaeocystis globosa virus	459984	826	0.2%
Polymorphum gilvum	4649365	18001	0.4%
Prochlorococcus marinus	1657990	296810	17.9%
Prochlorococcus phage MED4-213	180977	989	0.5%
Prochlorococcus phage P-GSP1	44945	1136	2.5%
Prochlorococcus phage P-SSM2	252407	300	0.1%
Prochlorococcus phage P-SSM7	182180	3175	1.7%
Prochlorococcus phage Syn1	191195	4277	2.2%
Prochlorococcus phage Syn33	174285	4976	2.9%
Propionibacterium acnes	2519002	2450832	97.3%
Pseudomonas fluorescens	6143950	46014	0.7%
Pseudomonas fulva	4920769	6930	0.1%
Pseudomonas mendocina	5072807	428758	8.5%
Pseudomonas phage LKA1	41593	13016	31.3%
Pseudomonas putida	6870827	408056	5.9%
Pseudomonas stutzeri	4709064	1620871	34.4%
Puniceispirillum phage HMO-2011	55282	5830	10.5%
Rahnella aquatilis	2766562	3868	0.1%
Ralstonia pickettii	1775243	10757	0.4%
Rhodanobacter denitrificans	4225490	10225	0.2%
Rhodobacter capsulatus	1935960	2742.5	0.2%
Rhodobacter sphaeroides	1461698	2004.33	0.3%
Rhodococcus erythropolis	3297806	18933	0.4%
Rhodococcus opacus	4229686.5	2595.5	0.1%
Roseobacter denitrificans	1081352.5	2596.25	1.8%
Roseobacter litoralis	1550624	6759	5.2%
Rothia mucilaginosa	2292716	311952	13.6%
Ruegeria pomeroyi	2300524	5500	0.2%
Serratia marcescens	5207023	16976	0.3%
Sphingobium japonicum	884972.4	11099.8	4.6%
Sphingomonas wittichii	1971748.7	14816	4.7%
Sphingopyxis alaskensis	1686856.5	34968.5	45.1%
Staphylococcus epidermidis	2454929	240828	9.8%
Stenotrophomonas maltophilia	4769156	56540	1.2%
Streptococcus suis	2028815	54495	2.7%
Streptococcus thermophilus	1796846	315319	17.5%
Sulfurimonas denitrificans	2201561	5508	0.3%
Synechococcus phage metaG-MbCM1	172879	132028	76.4%
Synechococcus phage S-MbCM100	170438	15176	8.9%
Synechococcus phage S-RIP1	44892	2746	6.1%
Synechococcus phage S-ShM2	179563	2432	1.4%
Synechococcus phage S-SKS1	208007	44401	21.3%
Synechococcus phage S-SM2	190789	2506	1.3%
Synechococcus phage S-SSM7	232878	450	0.2%
Thalassolituus oleivorans	3764053	24088	0.6%
Variovorax paradoxus	5626353	6430	0.1%
Zunongwangia profunda	5128187	52302	1.0%

Supplementary Table 4 CheckM statistics of genome bins

Bin name	Label	BACT completeness	BACT contamination	BACT strain-heterogeneity	genome size (bp)	# scaffolds
np-bin7-pan.bin7_1	*Legionella* sp.	100	0	0	2886101	95
outliers	Roseobacter fragments	n/a	n/a	n/a		
np-bin4	*Methylophaga* sp.	100	1.72	100	2541903	61
pangenome_np-bin7_2-pan.bin7_2_14	Diatom fragment	12.93	0	0	146965	19
pangenome_np-bin7_2-pan.bin7_2_2	*Polaribacter* sp.	55.02	10.19	5.26	1354003	166
pangenome_np-bin7_2-pan.bin7_2_3	Phage 1	0	0	0	977327	76
pangenome_np-bin7_2-pan.bin7_2_13	Phage 2	0	0	0	356493	22
pangenome_np-bin7_2-pan.bin7_2_5	*Erythrobacter* sp.	97.96	0	0	2558218	49
pangenome_np-bin7_2-pan.bin7_2_6	Roseobacter fragments	12.85	3.45	52	543659	49
np-bin5	*Escherichia coli*	96.55	0	0	4486681	170
pangenome_np-bin7_2-pan.bin7_2_9	Roseobacter fragments	10.34	0	0	184708	27
pangenome_np-bin7_2-pan.bin7_2_8	Phage 3	0	0	0	416069	27
pangenome_np-bin7_2-pan.bin7_2_0	Roseobacter fragments	19.2	4.55	11.11	2970438	403
np-bin6	Delta proteobacteria	46.16	1.72	100	746461	86
np-bin3	Gamma proteobacteria	29.86	1.72	0	1709939	224
pangenome_np-bin7_2-pan.bin7_2_4	NCLDV	1.41	0	0	545842	57
pangenome_np-bin7_2-pan.bin7_2_7	Roseobacter fragments	1.72	0	0	178918	28
np-bin2	*Novosphingobium* sp.	5.8	0	0	282480	44
pangenome_np-bin7_2-pan.bin7_2_12	Roseobacter frags + contamination	0	0	0	241080	24
pangenome_np-bin7_2-pan.bin7_2_10	Phage 4	0	0	0	490666	26
np-bin1	*Roseovarius* sp.	80.88	1.72	100	3387409	126
pangenome_np-bin7_2-pan.bin7_2_11	Phage 5	0	0	0	384320	24
pangenome_np-bin7_2-pan.bin7_2_1	*Roseobacter* sp.	74.61	0	0	4280636	394
np-bin8	Roseobacter fragments					
np-bin9	Roseobacter fragments					

Supplementary Table 5 Genome bin summaries

Genome bin	Annotation	Genome summary
NP1	*Roseovarius* sp.	The assembly is consistently annotated at *Roseovarius* based on ribosomal proteins and other conserved genes. The organism possesses microcompartments (ethanolamine) co-localized with polyamine uptake, which appears to be a novel system. Formate dehydrogenase suggests some C1 metabolism. This is the most abundant draft genome and is enriched in several aerosol samples.
NP2	*Novosphingobium* sp.	The assembly is consistently annotated as *Novosphingobium* but is an incomplete genome. It was very rare except at the beginning of the experiment.
NP3	Gamma proteobacteria 1	The genome is indicated as a SAR92/OM6 species, which possesses a Ni/Fe-dependent hydrogenase, a PQQ-dependent glucose dehydrogenase, and genes implicated in sulfur oxidization. It is relatively abundant throughout the experiment and generally evenly distributed between phases (bulk, SSML, and aerosol).
NP4	*Methylophaga sp* .	The assembly indicates a methylotroph, but unlike the closest reference genomes, it appears to lack NO reductase, dissimilatory nitrite reductase, and N_2O reductase. Instead it appears to have assimilatory nitrate and nitrite reductase. This species (bin) is most abundant early in the bloom.
NP5	*Escherichia coli* strain CAICE	The genome is annotated as *E. coli* but does not appear to have a Type III secretion system or many of the hallmarks of pathogenesis. It contains genes for capsular polysaccharide synthesis and multiple siderophore biosynthesis and uptake systems. The species is abundant in aerosols, particularly in the beginning of the experiment but is present throughout the experiment.
NP6	Delta proteobacteria 1	The genome indicates a basal deltaproteobacteria based on the phylogeny of r proteins.
NP8	*Roseobacter* fragments 1	This bin is annotated as a *Roseobacter* and is rare during the experiment.
NP9	*Roseobacter* fragments 2	This bin is very abundant particularly in the aerosol samples towards the end of the bloom. It contains a putative *rbcL* /*rbcS* gene cluster (carbon fixation via ribulose-bisphosphate carboxylase/oxygenase) and aerobic formate dehydrogenase-like protein (often can be hydrocarbon degrading).
7_1	*Legionella* sp.	Most ORFs have the highest amino acid similarity to *Legionella pneumophila* or *drancourtii* (70–80% identity) and represent a unique species, likely from a sister genus. The genome contains both Type II and Type IV secretion systems (Lsp and Dot/ICM respectively) as well as several ORFs with highest identity to eukaryotes. The genome also contains a Ni/Fe hydrogenase. The species is motile and compentent. It is most abundant early and found throughout the experiment.
7_2_0	*Roseobacter* fragments 6	This Roseobacter genome is most abundant in the water on day 8 of the experiment. It contains several elements of denitrification, including nitrite (Cu) reductase, nitric oxide reducatase. It is generally enriched in the aerosol phase.
7_2_1	*Roseobacter* sp.	The assembly yielded a moderately sized *Roseobacter* genome. It was abundant at the bloom peak, particularly in the aerosols. It contains a putative rbcL/rbcS gene cluster, in addition to nitrite reductase (Cu) and nitric oxide reductase. It may couple denitrification to carbon fixation.
7_2_2	*Polaribacter* sp.	The genome consistently suggests a basal Flavobacteria. It contains a Ni/Fe hydrogenase. It is mostly water-bourne and is most abundant during middle of the experiment.
7_2_3	Phage 1	The assembly is likely a Roseophage (phage of *Roseobacteria*) based on the similarity of auxillary metabolic proteins and the presence of clear orthologs for pelagiphage. The large size of the genome indicates this is probably not a single viral species and reflect many similar viral genomes.
7_2_4	NCLDV	This genome comprised a large bin with many hits to *Marseillevirus* .
7_2_5	*Erythrobacter* sp.	This species was abundant throughout the experiment, though most abundant towards the conclusion (Extended Data Fig. 5). It was generally found throughout all sample types.
7_2_6	*Rosebacter* fragments 3	This genome is abundant towards end of the experiment and was generally enriched in aerosols.
7_2_7	*Rosebacter* fragments 4	This assembly was rare throughout the experiment.
7_2_8	Phage 3	This assembly blooms at the end of the experiment in the 0.025 μm fraction. The abundance of *Roseobacter* and alpha-proteobacterial proteins, in addition to the clear phage genes (head, tail, baseplate wedge), indicate it a phage of these bacteria.
7_2_9	*Rosebacter* fragments 5	This assembly possibly contains a set of mobile genetic elements like plasmids. For example, much of the machinery for conjugal transfer (pilus, reverse transcriptase, resolvase, endonucleases, mate pair stabilization proteins), are present in this bin. It contains abundant *Roseobacter* elements and nitric oxide reductase and was present throughout the experiment.
7_2_10	Phage 4	Assembly indicates a phage with clear orthologs for tail, head, baseplate, but it is difficult to identify the host. A spike in abundance in the >3 μm fraction was followed by being very abundant in viral size fraction (0.025–0.2 μm) after the last two time points, with distributions in pretty much every substrate. This may indicate an early infection and burst event.
7_2_11	Phage 5	Assembly indicates a phage with clear orthologs for tail, head, baseplate, but it is difficult to identify the host. An abundance in the 0.025–0.2 μm fraction shifts to an increasing abundance in larger size fractions, and then reverts back to an increased amount in the 0.025–0.2 μm fraction indicating a possible transition from free phage, to infection of the host, and then to a burst releasing progeny phage.
7_2_13	Phage 2	Based on the similarity of auxillary metabolic proteins and the presence of clear orthologs for pelagiphage this assembly likely represents a phage for *Roseobacteria* . It is most abundant early in the experiment and is primarily absent from later time points.
7_2_14	Diatom fragment	This bin represents a diatom genome fragment. It includes several photosystem proteins, indicating it is likely a portion of the chloroplast genome, which is far more abundant than the nuclear chromosomes[7]. It is most abundant during the peak of the bloom in bulk, >3 μm fraction samples.

Supplementary Table 6 Analysis of draft genomes using k-mer based taxonomic profiling

Draft genome	Kraken Assignment	Assembly characteristics	Genome size (bp)	No. of Contigs	% complete
JCVI_CAICE_Erythrobacter_1	Erythrobacter litoralis		2558218	49	97.96
JCVI_CAICE_Novosphingobium_1	Novosphingobium aromaticivorans		282480	44	5.8
JCVI_CAICE_Methylophaga_1	Methylophaga nitratireducenticrescens	Many core methophaga genes	2541903	61	100
JCVI_CAICE_Gammaproteobacterium_1	Gammaproteobacterium HdN1	Basal gammaproteobacteria, SAR92	1709939	224	29.86
JCVI_CAICE_Escherichia coli_1	Escherichia coli		4486681	170	96.55
JCVI_CAICE_Legionella_1	Legionella pneumophila		2886101	95	100
JCVI_CAICE_Polaribacter_1	Polaribacter sp. MED152		1354003	166	55.02
JCVI_CAICE_Deltaproteobacterium_1	Candatus Babela massiliensis	Basal deltaproteobacteria	746461	86	46.16
JCVI_CAICE_Diatom_Fragment_1	Synechococcus sp. PCC_6312		146965	19	12.93
JCVI_CAICE_Roseobacter_1	Ruegeria pomeroyi		4280636	394	74.61
JCVI_CAICE_Roseovarius_1	Ruegeria pomeroyi		3387409	126	80.88
JCVI_CAICE_Roseobacter_fragments_1	-				
JCVI_CAICE_Roseobacter_fragments_2	Rhodobacter sphaeroides				
JCVI_CAICE_Roseobacter_fragments_3	Dinoroseobacter shibae		543659	49	12.85
JCVI_CAICE_Roseobacter_fragments_4	Bacillus infantis	Portion of roseobacter	178918	28	1.72
JCVI_CAICE_Roseobacter_fragments_5	Erythrobacter litoralis		184708	27	10.34
JCVI_CAICE_Roseobacter_fragments_6	Ruegeria pomeroyi	Missing core roseobacter genes	2970438	403	19.2
JCVI_CAICE_Roseobacter_fragments_7	Leisingera methylohalidivorans				
JCVI_CAICE_Roseobacter_fragments_contamination	Pseudomonas phage LKA1	Both alphaproteobacteria and roseobacter	241080	24	0
JCVI_CAICE_NCLDV_1	-		545842	57	1.41
JCVI_CAICE_Phage_1	Puniceispirillum phage HMO-2011	Large, T7-like	977327	76	0
JCVI_CAICE_Phage_2	Synecoccus phage ACG-2014c	Probable roseobacteria phage	356493	22	0
JCVI_CAICE_Phage_3	-		416069	27	0
JCVI_CAICE_Phage_4	Cotesia congregata bracovirus	Possible gammaproteobacteria phage	490666	26	0
JCVI_CAICE_Phage_5	-		384320	24	0

Taxonomic assignment of assembled metagenomics scaffolds using k-mer analysis (Kraken) with NCBI Refseq database. Assembled draft genomes identified utilizing APIS and manual curation. The assemblies were analyzed by Kraken to yield taxonomic assignments for comparison to raw read generated assignments. More complete genomes resulted in more accurate Kraken assignments. The diatom fragment derived from a chloroplast genome resulting in a cyanobacterial annotation in Kraken.

Supplementary Table 7 Population statistics for partially trimmed, fully trimmed, pooled, and day-resolved data sets

		AD - Value	P- Value	Skewness	Kurtosis	Mean	Std Dev	CV	Range	Minimum	Median	Maximum	N
Bacteria	ALL A:B	25.91	<0.0050	0.726	2.184	-0.019	0.374	-1985	3.91	-1.59	-0.05	2.33	3550
	ALL A:S	17.25	<0.0050	0.537	2.287	-0.070	0.357	-512	4.02	-1.63	-0.08	2.39	3550
	ALL AF	43.35	<0.0050	0.644	2.256	-0.044	0.367	-828	4.02	-1.63	-0.07	2.39	7100
	ALL S:B	-	<0.0050	0.328	4.848	0.057	0.243	423	3.31	-1.20	0.04	2.11	4260
Bacteria Trimmed	ALL A:B	4.47	<0.0050	0.945	1.765	0.097	0.501	516	3.31	-0.98	0.03	2.33	380
	ALL A:S	3.54	<0.0050	0.737	2.214	0.036	0.482	1352	3.99	-1.60	0.02	2.39	380
	ALL AF	7.70	<0.0050	0.848	1.981	0.066	0.492	742	3.99	-1.60	0.02	2.39	760
	ALL S:B	14.42	<0.0050	0.914	6.551	0.072	0.325	452	3.31	-1.20	0.03	2.11	456
Virus	ALL A:B	4.85	<0.0050	-0.491	-0.473	-0.010	0.651	-6508	3.40	-1.78	0.21	1.62	224
	ALL A:S	1.98	<0.0050	-0.300	0.257	-0.285	0.665	-233	3.95	-2.44	-0.23	1.51	223
	ALL AF	2.05	<0.0050	-0.380	-0.161	-0.147	0.672	-456	4.06	-2.44	-0.09	1.62	447
	ALL S:B	0.39	0.3826	-0.111	0.124	0.254	0.577	227	3.35	-1.67	0.22	1.68	263
Virus Trimmed	ALL A:B	1.58	<0.0050	0.219	-0.338	-0.008	0.827	-10183	4.16	-1.78	0.11	2.38	144
	ALL A:S	1.03	0.0101	-0.128	0.068	-0.410	0.701	-171	3.95	-2.44	-0.35	1.51	146
	ALL AF	0.47	0.250	0.196	0.045	-0.210	0.791	-376	4.82	-2.44	-0.21	2.38	290
	ALL S:B	0.30	0.5706	-0.204	0.034	0.276	0.629	228	3.38	-1.7	0.29	1.68	177
Bacteria A:B	day 8	12.53	<0.0050	1.353	4.175	-0.099	0.395	-400	3.34	-1.03	-0.16	2.31	710
	day 19	0.78	0.0434	0.122	1.290	0.092	0.436	471	3.91	-1.59	0.09	2.33	710
	day 24	0.87	0.0253	0.355	1.213	-0.160	0.288	-180	2.19	-1.10	-0.17	1.09	710
	day 28	5.46	<0.0050	0.508	3.723	-0.023	0.253	-1106	2.39	-1.02	-0.03	1.37	710
	day 34	8.40	<0.0050	0.619	0.765	0.095	0.393	413	2.62	-1.11	0.03	1.52	710
Bacteria A:S	day 8	6.08	<0.0050	0.902	2.142	-0.121	0.423	-348	2.96	-1.18	-0.17	1.78	710
	day 19	1.35	<0.0050	0.243	1.751	0.033	0.423	1264	4.02	-1.63	0.01	2.39	710
	day 24	0.68	0.0771	0.007	0.076	-0.137	0.282	-206	1.77	-0.97	-0.13	0.80	710
	day 28	1.47	<0.0050	0.331	1.595	-0.060	0.247	-412	1.97	-0.96	-0.06	1.01	710
	day 34	6.62	<0.0050	0.340	1.600	-0.064	0.350	-550	2.78	-1.60	-0.09	1.19	710
Bacteria S:B	day 0	7.93	<0.0050	-0.861	0.995	0.090	0.343	381	2.14	-1.20	0.14	0.93	710
	day 8	13.70	<0.0050	1.258	6.142	0.022	0.185	822	1.76	-0.66	0.01	1.11	710
	day 19	5.46	<0.0050	1.154	5.543	0.059	0.122	206	1.34	-0.34	0.05	1.00	710
	day 24	8.48	<0.0050	0.317	2.347	-0.023	0.179	-774	1.53	-0.68	-0.02	0.85	710
	day 28	*	<0.0050	1.933	16.100	0.037	0.160	429	2.10	-0.50	0.03	1.61	710
	day 34	3.81	<0.0050	0.286	2.932	0.159	0.332	209	3.11	-1.00	0.17	2.11	710
Virus A:B	day 8	1.85	<0.0050	0.049	-1.483	-0.230	0.823	-358	2.96	-1.78	-0.29	1.18	43
	day 19	1.64	<0.0050	1.726	4.575	-0.227	0.507	-224	2.59	-0.97	-0.25	1.62	44
	day 24	1.91	<0.0050	-0.244	-1.519	-0.322	0.648	-201	2.12	-1.54	-0.08	0.58	45
	day 28	2.10	<0.0050	-2.931	14.059	0.303	0.351	116	2.24	-1.45	0.31	0.79	44
	day 34	2.40	<0.0050	0.211	4.307	0.881	0.548	62	3.32	-0.94	0.86	2.38	41
Virus A:S	day 8	2.88	<0.0050	-0.020	-1.703	-0.417	1.168	-280	3.95	-2.44	-0.17	1.51	44
	day 19	0.67	0.077	0.526	-0.100	-0.224	0.474	-212	2.01	-1.20	-0.27	0.81	45
	day 24	0.97	0.0135	0.398	0.170	-0.483	0.538	-111	2.27	-1.35	-0.36	0.92	46
	day 28	0.29	0.6091	-0.100	-0.599	-0.214	0.376	-176	1.61	-1.04	-0.21	0.57	44
	day 34	0.91	0.019	0.695	1.589	-0.080	0.367	-457	1.82	-0.87	-0.10	0.95	44
Virus S:B	day 0	0.31	0.5526	-0.431	-0.023	-0.221	0.542	-245	2.43	-1.67	-0.22	0.76	46
	day 8	0.79	0.0362	-0.654	0.244	0.183	0.490	268	2.30	-1.26	0.29	1.04	42
	day 19	0.29	0.5923	0.333	0.720	0.000	0.327	-3.4×10^{18}	1.58	-0.71	0.01	0.87	45
	day 24	0.41	0.3244	-0.372	-0.447	0.172	0.352	204	1.49	-0.68	0.22	0.81	45
	day 28	0.60	0.1128	-0.289	1.711	0.532	0.497	93	2.77	-1.12	0.50	1.65	46
	day 34	1.68	<0.0050	-1.295	1.716	0.951	0.414	44	1.96	-0.28	1.03	1.68	39

Normality is tested by the Anderson–Darling (AD) normality test, skewness, kurtosis (excess), and inspection of histograms. For the AD test, p-values below 0.05 demonstrate data is significantly non-normal. Departure from zero indicates increasing non-normality in skewness and kurtosis values.

Supplementary Table 8 Percent of population in bacterial and viral reads in >3 μm, 3–0.2 μm, and 0.2–0.025 μm size fractions in bulk, SSML, and aerosol samples from read-based k-mer sequence analysis

	Bulk > 3 μm	SSML > 3 μm	Aerosol > 3 μm	Bulk 3 - 0.2 μm	SSML 3 - 0.2 μm	Aerosol 3 - 0.2 μm	Bulk 0.2 - 0.025 μm	SSML 0.2 - 0.025 μm	Aerosol 0.2 - 0.025 μm
Bacteria	91.45	91.13	92.50	95.13	95.91	96.49	91.49	83.01	92.25
Viruses	7.28	7.72	6.86	4.27	3.63	3.03	8.00	16.15	7.18

3. Supplementary Notes

Supplementary Note 1: Bloom dynamics

Chlorophyll *a* peaked at 21.7 μg L^{-1} for bloom 1 and 30.0 μg L^{-1} for bloom 2 (Fig. 1, Supplementary Fig. 1). Major phytoplankton blooms at Scripps Pier generally have chlorophyll *a* levels in the range of 18 – 219 μg L^{-1} [1], placing this on the low end of natural major bloom from the same source water. Bacteria counts were found to be in the range of 0.65 X $10^6 \pm$ 0.01 X 10^6 to 3.98 X $10^6 \pm$ 0.02 X 10^6 cells mL^{-1} in bulk water, 0.36 X $10^6 \pm$ 0.01 X 10^6 to 3.34 X $10^6 \pm$ 0.03 X 10^6 cells mL^{-1} in SSML, and 0.09 X $10^7 \pm$ 0.06X 10^7 to 5.83 X $10^7 \pm$ 0.58 X 10^7 cells m^{-3} in aerosol. Oceanic values for bacteria range from 10^4 to 10^6 cells mL^{-1} [2], making the values here on the high end of ocean concentrations. However, considering bacterial abundance increases linearly with chlorophyll [3] and under certain conditions oceanic concentrations have been found to up to 10^8 cells mL^{-1} [4], these values seem especially relevant. Aerosol bacteria concentrations are estimated to be ~10^2 to 10^4 cells m^{-3} [5]. Virus abundances were found to be 1 X 10^7 to 8 X 10^7 cells mL^{-1} in bulk and SSML and 0.3 X 10^7 to 7.7 X 10^7 cells m^{-3} in aerosol which is ~10-fold higher than water bacteria concentrations and approximately the same as bacterial concentrations in aerosol.

Supplementary Note 2: Genomic data analysis

DNA yields. DNA recovery and estimated original concentrations in native samples are given in Supplementary Table 1. DNA abundance was determined by Qubit™ (ThermoFisher).

Metagenomic sequencing. A total of 625 million reads were trimmed and quality filtered generating on average 3.0 million sequence reads with lengths of 2 x 142 bp and GC content of 48% (Supplementary Table 2). Taxonomic profiling generated a total of 700 bacterial, 10 archaeal and 28 viral species in samples from the 0.02–3 μm size fraction after data filtering. The >3 μm and 0.025–0.2 μm fraction were also trimmed similarly to generate 613 species (576 bacteria, 8 archaea, 29 viruses) and 627 species (585 bacteria, 7 archaea, 35 viruses), respectively. The viral species from the three fractions were pooled to yield 46 viral species.

Coverage analysis. 198 bacteria and 46 viral species identified using Kraken were further examined for genomic coverage. Scaffolds from species' assignments were examined against published genomes (Supplementary Table 3). This represented 28% of the species above abundance thresholds. Species below 0.1% spatial coverage were removed resulting in a coverage trimmed set of 76 bacterial and 30 viral species.

Genomic assembly and annotation. Sixty-two metagenomes resulted from assembly of shotgun reads. k-mer based binning (Vizbin) isolated seven bins (Np-bin1, 2, 3, 4, 5, 6, 8) and a mosaic bin (bin7). The remaining bin7 was a mosaic of genomes and thus was examined using sample-specific sequencing coverage vectors and hierarchical clustering, generating a further 14 bins. Completeness of bins was assessed using CheckM (Supplementary Table 4).

Genome bins, while mostly not complete, were recovered for Bacteria, Eukarya, and multiple viral lineages. The completeness appears to be mostly due to more conserved regions of the genome fragmenting during the secondary co-assembly, which did decrease large contigs. The low completeness (CheckM) for several genomes rather large in size is consistent with this. Annotation of genomes was performed by APIS and manual curation[6]. Summaries of genome bins are given in Supplementary Table 5. Additionally, the resulting draft genomes were run through read-based pipeline utilizing Kraken to detect similarity between the annotations of the different approaches. There was good agreement between annotations when genome coverage was adequate.

4. Supplementary References

1. Kim, H., Miller, A. J., McGowan, J. & Carter, M. L. Coastal phytoplankton blooms in the Southern California Bight. *Prog. Oceanogr.* **82,** 137–147 (2009).
2. Whitman, W. B., Coleman, D. C. & Wiebe, W. J. Prokaryotes: the unseen majority. *Proc. Natl. Acad. Sci. U. S. A.* **95,** 6578–6583 (1998).
3. Bird, D. F. & Kalff, J. Empirical Relationships between Bacterial Abundance and Chlorophyll Concentration in Fresh and Marine Waters. *Can. J. Fish. Aquat. Sci.* **41,** 1015–1023 (1984).
4. Turley, C. M. & Mackie, P. J. Biogeochemical significance of attached and free-living bacteria and the flux of particles in the NE Atlantic Ocean. *Mar. Ecol. Prog. Ser.* **115,** 191–204 (1994).
5. Burrows, S. M., Elbert, W., Lawrence, M. G. & Pöschl, U. Bacteria in the global atmosphere – Part 1: Review and synthesis of literature data for different ecosystems. *Atmos. Chem. Phys.* **9,** 9263–9280 (2009).
6. Dupont, C. L. *et al.* Functional tradeoffs underpin salinity-driven divergence in microbial community composition. *PLoS One* **9,** (2014).
